# Identifying Inputs to Visual Projection Neurons in *Drosophila* Lobula by Analyzing Connectomic Data

**DOI:** 10.1523/ENEURO.0053-22.2022

**Published:** 2022-04-21

**Authors:** Ryosuke Tanaka (田中涼介), Damon A. Clark

**Affiliations:** 1 Interdepartmental Neuroscience Program, Yale University, New Haven, CT 06511; 2 Department of Molecular Cellular and Developmental Biology, Yale University, New Haven, CT 06511; 3 Department of Physics, Yale University, New Haven, CT 06511; 4 Department of Neuroscience, Yale University, New Haven, CT 06511

**Keywords:** connectome, *Drosophila*, feature detectors, lobula, visual processing, visual projection neurons

## Abstract

Electron microscopy (EM)-based connectomes provide important insights into how visual circuitry of fruit fly *Drosophila* computes various visual features, guiding and complementing behavioral and physiological studies. However, connectomic analyses of the lobula, a neuropil putatively dedicated to detecting object-like features, remains underdeveloped, largely because of incomplete data on the inputs to the brain region. Here, we attempted to map the columnar inputs into the *Drosophila* lobula neuropil by performing connectivity-based and morphology-based clustering on a densely reconstructed connectome dataset. While the dataset mostly lacked visual neuropils other than lobula, which would normally help identify inputs to lobula, our clustering analysis successfully extracted clusters of cells with homogeneous connectivity and morphology, likely representing genuine cell types. We were able to draw a correspondence between the resulting clusters and previously identified cell types, revealing previously undocumented connectivity between lobula input and output neurons. While future, more complete connectomic reconstructions are necessary to verify the results presented here, they can serve as a useful basis for formulating hypotheses on mechanisms of visual feature detection in lobula.

## Visual Abstract

**Figure:**
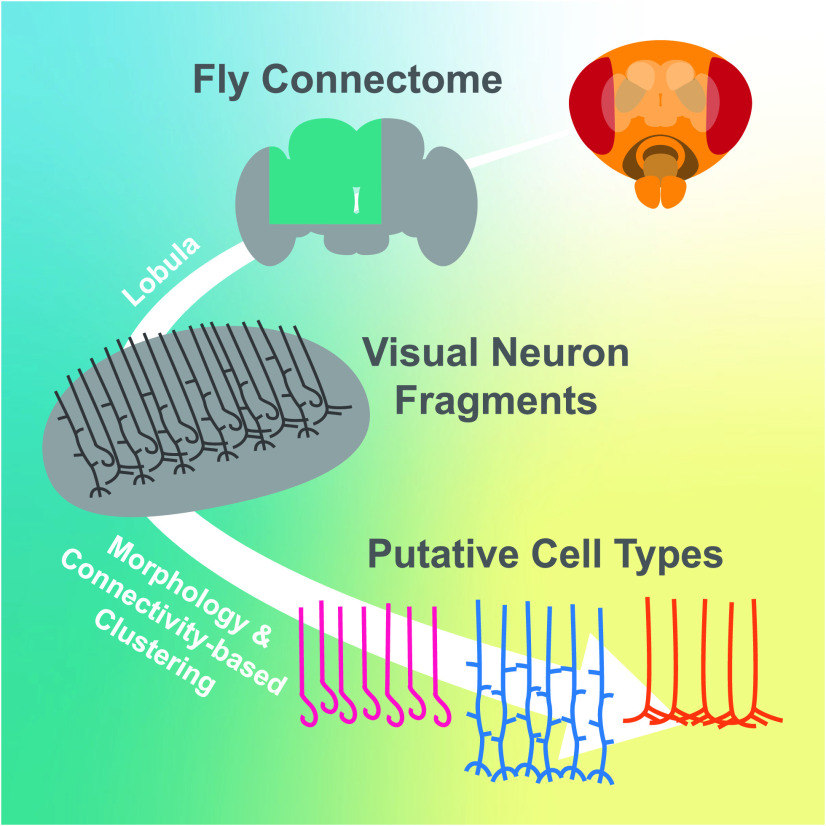

## Significance Statement

The lobula is a structure in the brain of the fly that is thought to be dedicated to detecting behaviorally relevant visual objects, such as other flies or predators. However, how the input and output neurons of the lobula are connected, key information to understand its neural computations, has remained mostly unknown. Here, we analyzed fragmented lobula input neurons in a recently published electron microscopy (EM)-based connectomic database. We categorized them into putative cell types, based on their connectivity and morphology. Our analysis identifies previously unknown connectivity between input and output neurons of the lobula, which can guide future physiological studies into the mechanisms by which output neurons of the lobula acquire their selectivity for specific types of visual objects.

## Introduction

During the past decade, studies of the visual system of the fruit fly *Drosophila melanogaster* have generated an exquisitely detailed picture of how various visual computations are achieved by circuits of neurons (Schnaitmann et al., 2018; Yang and Clandinin, 2018; de Andres-Bragado and Sprecher, 2019; Cheong et al., 2020). In addition to the relative numerical simplicity of the fly brain (Raji and Potter, 2021) and its sophisticated genetic tools to monitor and manipulate specific cell types (Kitamoto, 2001; Pfeiffer et al., 2010; Jenett et al., 2012; Kazama, 2015; Tirian and Dickson, 2017; Simpson and Looger, 2018), a major contributor to this rapid progress has been electron microscopy (EM)-based connectomes (Meinertzhagen, 2016, 2018). For example, dense EM reconstruction of circuitry surrounding the elementary motion detecting neurons in the *Drosophila* brain, T4 and T5 (Takemura et al., 2013, 2015, 2017; Shinomiya et al., 2014, 2019), have guided functional studies by providing strong constraints on neural computations in T4 and T5 (Behnia et al., 2014; Strother et al., 2017; Borst, 2018; Agrochao et al., 2020; Zavatone-Veth et al., 2020; Kohn et al., 2021; Ramos-Traslosheros and Silies, 2021), as well as by discovering previously undocumented circuit elements (Behnia et al., 2014; Ammer et al., 2015; Serbe et al., 2016; Strother et al., 2017; Meier and Borst, 2019). Similarly, EM reconstruction of early visual neuropils led to the discovery of a novel pathway for color vision (Takemura et al., 2008), which was later functionally confirmed to be contributing to spectrally-sensitive behaviors (Gao et al., 2008).

**Figure 1.**
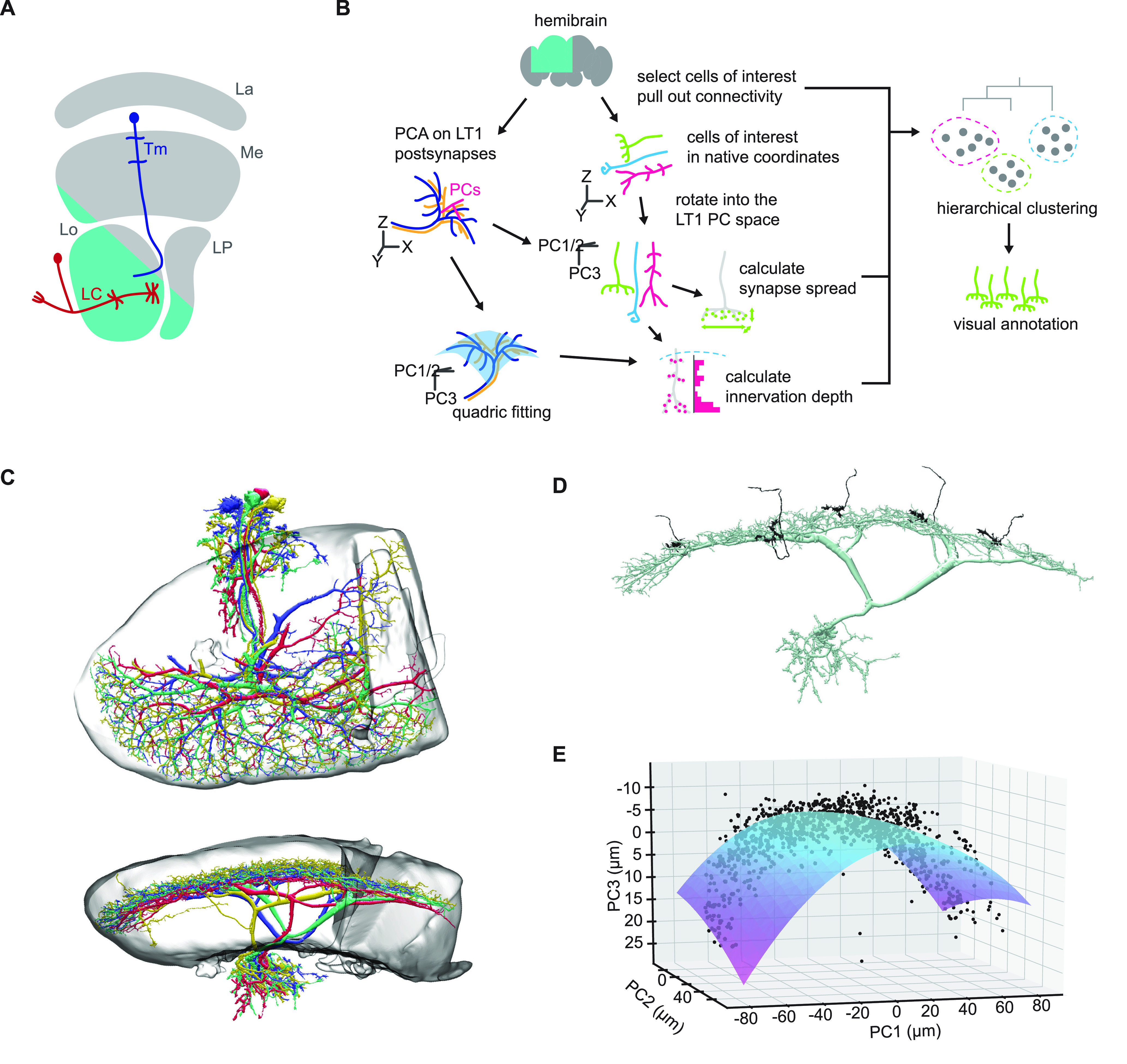
An overview of the present study. ***A***, A schematic of the *Drosophila* optic lobe. The optic lobe consists of four neuropils, namely, lamina (La), medulla (Me), lobula (Lo), and lobula plate (LP). The lobula houses projection neurons such as lobula columnar (LC) and lobula tangential (LT), which send axon terminals to the central brain and are thought to be responsible for detecting various visual features for approach and avoidance behaviors. Neurons connecting the medulla and lobula, such as transmedullar (Tm) neurons, are likely providing major synaptic inputs into LCs and LTs, but their connectivity remains largely unknown. The teal shaded region roughly indicates the volume reconstructed in the hemibrain dataset. ***B***, A schematic of the analysis pipeline. Small lobula intrinsic neurons in the hemibrain dataset were selected as cells of interest, and their connectivity to labeled downstream neurons were tabulated. The candidate cells were then rotated into the principal component (PC) space of LT1 postsynapses, which we used as a proxy for layer 2 of the lobula, from which we derived normal and tangential axes of the lobula layers. We then quantified the morphology of the cells of interest by synapse spread in the PC space and by the histogram of innervation depth relative to a parabolic surface fit to the LT1 synapses. Finally, we applied hierarchical clustering to the concatenated feature matrix, and visually annotated the resulting clusters. ***C***, The morphology of reconstructed LT1 neurons, viewed from (top) normal and (bottom) tangential directions, visualized alongside with the boundary of the lobula neuropil. The dendrites of a single LT1 neuron cover the entire tangential extent of the lobula, rather than tiling the lobula as a population. The dendrites of LT1 form a single thin layer around layers 2 and 3 of the lobula. ***D***, The morphology of a single LT1 neuron (green), with several cells of interest in cluster 1 (see also Figs. 4, 6), viewed tangentially. ***E***, The parabolic surface (blue) fit to the LT1 presynapses (black). For clarity, only a random 10% of the total LT1 presynapses are visualized.

In addition to motion and color, the visual system of flies is equipped with neurons tuned to a variety of visual features. Recent studies have shown that a series of columnar output neurons of the lobula (Fig. 1*A*) collectively named lobula columnar (LC) and lobula plate-LC (LPLC) neurons are tuned to visual features resembling conspecifics and predators, and their activity can induce a variety of behavioral responses, depending on types (Panser et al., 2016; Wu et al., 2016; Keleş and Frye, 2017; Klapoetke et al., 2017; von Reyn et al., 2017; Ribeiro et al., 2018; Ache et al., 2019; Städele et al., 2020; Tanaka and Clark, 2020, 2021; Sten et al., 2021). Several of these neurons are thought to achieve their selective visual tuning by pooling the feature selectivity of their presynaptic partners (Klapoetke et al., 2017; Keleş et al., 2020; Tanaka and Clark, 2020, 2021). However, the presynaptic partners of these LC and LPLC neurons remain largely unknown. This is because of the lack of connectomic reconstruction encompassing the lobula and its upstream neuropils. For example, a recent connectomic dataset used to analyze the motion detection circuitry included most of the visual system, but the reconstruction was focused on T4 and T5 and their inputs, leaving out the most of the lobula circuitry (Shinomiya et al., 2019). Similarly, community-driven tracing and proofreading effort of the full adult fly brain (FAFB) dataset (Zheng et al., 2018) is still in progress. While the densely-reconstructed hemibrain dataset contains almost the entire lobula, since it lacks almost the entire medulla, neurons providing inputs into the lobula [e.g., transmedullar (Tm) neurons) are fragmented and unlabeled; Scheffer et al., 2020].

However, recent studies have shown that close inspection of these fragmented axon terminals of input neurons into the lobula in the hemibrain dataset can still offer potentially useful information about the lobula circuitry (Tanaka and Clark, 2020, 2021). In the present study, we perform clustering analyses on those fragmented terminals based on their connectivity and morphology, defining putative input cell types for intrinsic and projection neurons of the lobula, including LC and LPLC neurons. This analysis comes with the caveat that it is difficult to conclusively identify cell types based on their axonal morphology alone and thus needs to be complemented by a more comprehensive connectome encompassing the entire optic lobe. Even so, the results presented here provide a previously unknown putative connectivity to physiologists and psychophysicists interested in visual processing in the lobula.

## Materials and Methods

The goal of present analysis is to categorize unlabeled putative columnar neurons in lobula into cell types, and examine their connectivity to downstream neurons. Typically, a columnar visual neuron type consists of many cells sharing stereotypic connectivity and morphology tiling the visual space. Thus, the problem boils down to grouping neurons by their individual connectivity and morphology while discounting their exact spatial location. To this end, we performed an agglomerative clustering on their connectivity and morphology. A schematic of the analysis pipeline is shown in Figure 1*B*.

### Code accessibility

The code to perform the analysis presented in the paper is freely available online at https://github.com/ClarkLabCode/LobulaClustering. The code is available as Extended Data 1.

10.1523/ENEURO.0053-22.2022.ed1 Extended Data 1 The code to perform the analysis. Download Extended Data 1, ZIP file.

### Inclusion criteria

Cells without cell type assigned in the hemibrain 1.2.1 dataset that had synapses only in the lobula and had >50 and fewer than 500 total synaptic sites were selected. The upper bound of 500 synaptic sites was comparable to the size of the smallest LC neurons (e.g., LC12) or lobula intrinsic neurons (e.g., Li13). Cells with fewer than 50 synaptic sites were excluded because they were difficult to classify reliably because of the small amount of connectivity data available. We refer to these cells as “cells of interest.” A total of 7465 cells of interest met the above criteria and were incorporated to the analyses.

### Connectivity

For each cell of interest, we counted the number of synapses it has onto labeled cell types in the lobula. Connections to multiple cells of a single cell type were summed together to generate a single feature, rather than being treated separately. This was intended to facilitate grouping of cells belonging to the same cell types with a shared pattern of connectivity regardless of their retinotopic location. A total 237 labeled cell types were found to be downstream of at least one of the cells of interest in the present analysis.

### Morphology

To summarize features of the axonal morphology of the cells of interest, we calculated (1) the standard deviation of synapse position along the two tangential axes and the normal axis of the lobula layers, as well as (2) the histogram of synapses along the depth of the lobula. The native coordinate system of the hemibrain dataset is a Cartesian system where X points left, Y points anteriorly, and Z points ventrally (Scheffer et al., 2020). To identify the normal and tangential axes of the lobula layers, we first performed principal component analysis (PCA) on the postsynapses of LT1 neurons. LT1 is a tangential neuron with wide, dense, monostratified dendrites in layer 2 of the lobula (Fischbach and Dittrich, 1989; Fig. 1*C*,*D*). The hemibrain dataset contains four LT1 neurons, each covering the entire tangential extent of the lobula. The resulted first two principal component (PC) axes were approximately aligned to the ventrolateral and anteroposteior axes of the lobula, respectively, and the third PC axis was normal to that plane. Then, for each cell of interest, we calculated the standard deviations of the distributions of its presynapses along the three PC axes, which quantified tangential extents and vertical diffuseness of its axon terminal. We refer to these three features as “synapse spread” hereafter.

Next, we aimed to calculate the depth of each synapse along the layers of the lobula. Since the layers of the lobula are curved, the raw position of the synapses along the third PC axis do not accurately correspond to their layer affiliation. To solve this issue, we first fit a parabolic surface:

$$\hat{z}=a_{0}+a_{1}x+a_{2}y+a_{3}x^{2}+a_{4}y^{2}+a_{5}xy,$$

to the postsynapses of the LT1 neurons with the least-square method, where x and y represent position along the first and second PC axes (μm), $\hat{z}$ represents the predicted position along the third PC axis (μm), and $a_{i}$ are coefficients (Fig. 1*E*). The fit coefficients were as follows: a_0_ = −5.21, a_1_ = 4.04 × 10^−3^, a_2_ = 4.04 × 10^−3^, a_3_ = 3.17 × 10^−3^, a_4_ = 7.65 × 10^−4^, a_5_ = −1.22 × 10^−3^. The goodness of fit was R^2^ = 0.744. We then calculated the depth of each synapse relative to layer 2 as $z-\hat{z}$, where z represents the true position of the synapse along the third PC axis. Note that this approximates the lobula layers to be parallel to each other, rather than concentric curved surfaces. Deviations from this approximation will be most severe far from the center of this PC coordinate system. For each cell of interest, we counted the number of presynapses in 5 μm wide bins ranging from −20 to +50 μm of relative depth, resulting in 14 features. This range covered the entirety of the lobula. We refer to these features as “innervation depth” in the following. Similar analyses of innervation depth have been performed previously on medulla neurons (Bausenwein et al., 1992; Zhao and Plaza, 2014). We validated that the synapse spread and innervation depth calculated this way appropriately reflect the neuronal morphology by applying the same analysis to postsynapses on the dendrites of multiple LC types with well characterized morphology (Wu et al., 2016).

### Agglomerative clustering on cells of interest

We concatenated the connectivity, synapse spread, and smoothed innervation depth feature matrices into a single feature matrix, and performed an agglomerative clustering with Ward’s variance minimization method (Ward, 1963) with SciPy (Virtanen et al., 2020). Before being concatenated, connectivity, synapse spread, and innervation depth matrices were, respectively, normalized by its total dispersion (sum of variances) and then weighted by the factors of 5, 1, and 3, respectively. This weighting reflected our relative confidence on the accuracy of the three feature sets. We set the number of clusters to be 40, which is approximately comparable to the number of Tm and TmY cell types reported in Fischbach and Dittrich (1989). The complete results of the clustering analysis are presented in Extended Data 2.

10.1523/ENEURO.0053-22.2022.ed2 Extended Data 2 The complete results of the clustering analysis. Download Extended Data 2, ZIP file.

#### Annotation and visualization

Next, we visualized the cells of interest in each resultant clusters on the neuPrint website (Clements et al., 2020a) and compared their terminal morphology with existing anatomic literature (Fischbach and Dittrich, 1989) to determine cell type identity. We also performed a nonlinear dimensionality reduction on the concatenated feature matrix with uniform manifold approximation (UMAP; McInnes et al., 2018) for visualization.

### Quantification of connectivity between cells of interest and visual projection neurons

Lastly, we analyzed the connectivity between the 40 clusters of cells of interest and 21 LC and LPLC types (Wu et al., 2016). For each LC or LPLC type, we calculated relative number of synapses they receive as a population from each of the 40 clusters. Additionally, an agglomerative clustering was performed on the relative connectivity matrix between the 40 clusters and the 21 LC/LPLCs.

## Results

### The synapse spread and innervation depth features capture the dendritic morphology of LC neurons

**Figure 2.**
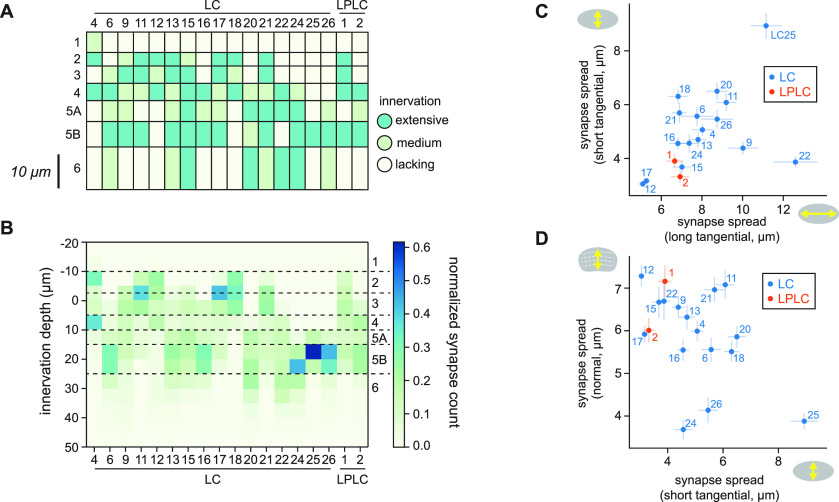
Validation of morphologic features. ***A***, A diagram showing innervation patterns of LC and LPLC neurons in the lobula, based on a previously published data (Wu et al., 2016, see their Fig. 5). ***B***, Innervation depth features of the identical set of LC and LPLC neurons as in ***A***. Innervation depth were averaged across all the cells in each cell type and then normalized within each type. The horizontal dotted lines indicate the approximate boundaries between layers inferred by comparing ***A***, ***B***. ***C***, ***D***, Mean synapse spread of LCs and LPLCs along the long and short tangential axes and the normal axis, with SEM.

To gain confidence in our quantification of cellular morphology, we first calculated innervation depth and synapse spread features for the lobula postsynapses of 18 LC and LPLC projection neuron types with well characterized morphology (LC4, 6, 9, 11–13, 15–18, 20–22, 24–26, LPLC1 and 2; Wu et al., 2016; Fig. 2). Figure 2*A*,*B* shows previously reported light microscopy-based innervation patterns of LCs and LPLCs (Wu et al., 2016; Fig. 2*A*) side by side with their connectome-based innervation depth feature (Fig. 2*B*). The patterns of innervation captured by two methodologies agreed well. For example, the bistratified dendrites of LC4 in the layers 2 and 4 are clearly visible from the connectome-based innervation depth features (Fig. 2*A*,*B*, the leftmost column). The synapse spread of LCs and LPLCs along the two tangential and the normal axis of the lobula are shown in Figure 2*C*,*D*. These features also captured well the known morphology of these neurons. For example, LC25 and 12, which, respectively, had large and small synapse spread along the both tangential dimensions (Fig. 2*C*) have been previously shown to have large and small isotropic dendrites (Wu et al., 2016, see their Fig. 6). LC22, which had large synapse spread along the long tangential axis of the lobula but small spread along the short axis, indeed has anisotropic, narrow dendrites (Wu et al., 2016, see their Fig. 6). In a similar vein, along the normal axis, vertically diffuse neurons such as LPLC1 and LC15 (Wu et al., 2016, see their Fig. 5) had large synapse spread, whereas monostratified LC25 had small spread (Fig. 2*D*). Overall, these results indicate that the two morphologic features we calculated accurately capture the morphology of neurites in the lobula neuropil.

### Agglomerative clustering identifies clusters with homogeneous connectivity and morphology

**Figure 3.**
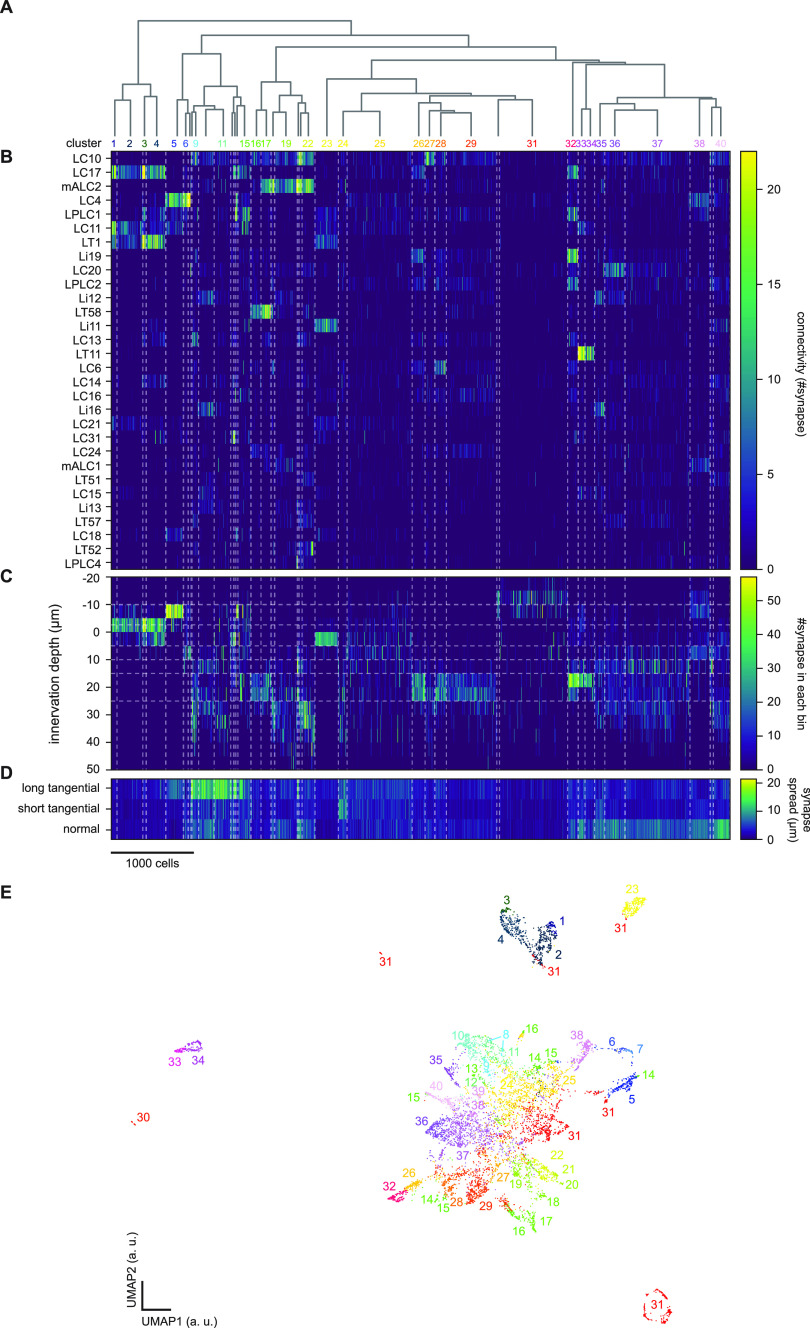
Agglomerative clustering of unlabeled lobula neurons. ***A***, The dendrogram of the clusters, with cluster labels. Only clusters with >50 cells are labeled for visibility. ***B***, Connectivity between the cells of interest, sorted by cluster affiliation, and the top 30 labeled lobula neuron types with the largest number of synapses from the cells of interest. The white dotted lines delineate clusters, throughout ***B–D***. ***C***, Innervation depth of the cells of interest, sorted by cluster affiliation. The horizontal dotted lines indicate approximate layer boundaries, as in Figure 2*B*. ***D***, Synapse spread of the cells of interest along the tangential and normal axes of the lobula. ***E***, The cells of interest on the UMAP embedding space, based on the normalized, weighted, and concatenated features. Clustering was performed directly on the feature matrix, so that the UMAP embedding is simply for visualization. a. u.: arbitrary unit.

**Figure 4.**
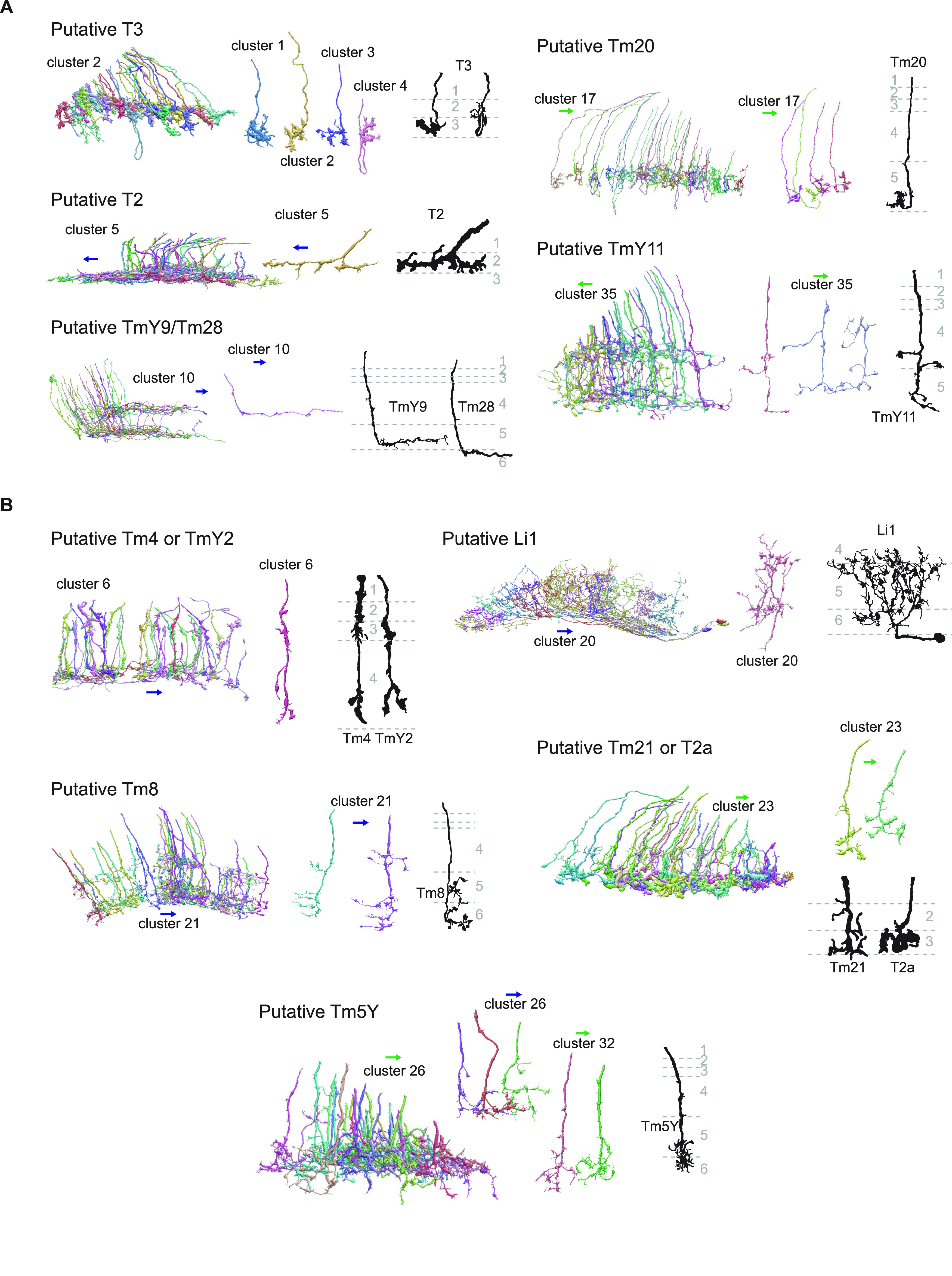
Example clusters with putative morphologic matches. ***A***, Clusters morphologically identified with known cell types with the highest confidence, labeled as A in Table 1. For each putative cell type, (left) a population of reconstructed neurons in the corresponding clusters viewed tangentially, (middle) individual examples of cells in the clusters, and (right) Golgi staining from Fischbach and Dittrich (1989) are shown. ***B***, The same as ***A***, but for clusters with less confidence (labeled as B in Table 1). The green and blue arrows, respectively, point toward anterior and ventral directions.

The results of the clustering are summarized in Figures 3, 4 and Table 1. The detailed analyses of each cluster will be presented in the final part of Results. Clustering successfully extracted groups of cells with similar connectivity and morphology, as can be seen from the block-like appearance of the feature matrices (Fig. 3*B–D*). Additionally, we visualized the cells of interest on the two-dimensional nonlinear embedding by UMAP (Fig. 3*E*). Overall, the embedding agreed well with the hierarchical clustering, since neighboring clusters are often neighbors in the UMAP space. This was not without exceptions, such as clusters 15, 16, 29, and 31, which had clear substructures on the UMAP embedding. Table 1 summarizes the morphology, connectivity, and putative cell type affiliation of all identified clusters. Example clusters that we identified with previously documented types of neurons with reasonable confidence are shown in Figure 4. The input columnar cell types we identified most confidently included T3, T2, TmY9/Tm28, Tm20, and TmY11 (Fig. 4*A*).

**Table 1:** Summary of clustering on the cells of interest

#	Homogeneity	Putative cell type	Confidence	Layers	Morphology	Connectivity	Related to
1	A	T3	A	2, 3	Small, crumpled, sometimes with loops	LC17, LC11, LT1	#2–4
2	A	T3	A	2, 3	Small, crumpled, sometimes with loops	LC17, LC11, LT1	#1, 3, 4
3	A	T3	A	2, 3	Small, crumpled, sometimes with loops	LC17, LC11, LT1, LPLC1	#1, 2, 4
4	A	T3	A	2, 3	Small, crumpled, sometimes with loops	LC17, LC11, LT1, LPLC1	#1–3
5	A	T2	A	2	T shaped, spiny	LC4, LC11, LC18, Li15, LPLC1	
6	B	Tm4 or TmY2	B	2, 4	Small, vertical, Y-shaped ends, swelling in Lo2	LC4, LPLC1	
7	C	mixture	NA	2, 4		LC4, LC12	
8	A	?	NA	6	Wide field, lobula intrinsic	LC10, LC20, mALC2, LT52, Li12	
9	A	?	NA	5B	Anisotropic, bent dorsally	LC10, LC13, Li19, LC6, LT57	
10	B	TmY9 or Tm28	A	4, 5B	Linear, L-shaped, optional branching and layer-switching	LC10, Li16, Li12, LC15, Li13	
11	C	mixture	NA	4–6		LC10	
12	A	Tm19	C	3–5B	Meandering, large secondary branch	LT87, LC31, LC17	
13	A	Tm19	C	3–5B	Meandering, large secondary branch	LT87, LC31, LC17	
14	C	mixture	NA	2–5A		LPLC1	#5
15	C	mixture	NA	2–5B		LPLC1	
16	A	Tm11	C	5B	Thin, knobby, posteriorly oriented	LT58, LC10, LC24	
17	A	Tm20	A	5B	Thick, compact	LT58, mALC2, LC10, LC24	#29
18	A	?	NA	5B, 6	Short branches, knobby endings	mALC2, LPLC2, LT58, LC22, LC6	
19	A	TmY10	C	5A–6	Somewhat vertically diffuse, small protrusions in shallower layers	mALC2, LC10, mALC1, Li12	
20	A	Li1	B	4, 5A, 6	Lobula intrinsic, bistratified, knobby	mALC2, LPLC4, LC10, Li13, LT36	
21	A	Tm8	B	5A, 6	Bistratified, dorsally oriented multiple branches	mALC2, LC10, LT57, LC13, LT52	
22	B	?	NA	5A, 6	Thick, or thin and knobby (two cell types)	mALC2, LC10, LT52, LT63, LT51	
23	A	T2a or Tm21	B	3	Monostratified, small, crumpled	Li11, LT1, LC11, LPLC1, LC21	
24	C	mixture	NA	diffuse		mALC2	
25	C	mixture	NA	diffuse		LC4	
26	A	Tm5Y	B	5A/B	Thick, ventrally oriented, monostratified, small protrusions in shallower layers	Li19, LC17, LT79, LPLC2, LC6	#32
27	B	?	NA	5B	Thin, monostratified, knobby endings	LC10	#29
28	A	?	NA	5A/B	Thin, monostratified, knobby endings	LC6, LPLC2, LC10, Li19, LC16	
29	B	?	NA	5A/B		LC10, LC16	#17, 27
30	A	Tm9	A	1	Small, crumpled	CT1	
31	C	mixture	NA	1	T5 and its inputs		
32	A	Tm5Y	B	5A/B	Thick, ventrally oriented, monostratified, small protrusions in shallower layers	Li19, LC17, LT79, LPLC2, LPLC1	#26
33	A	Tm5	B	5A/B	Straight, small protrusion in a shallower layer	LT11, LC25, LC11, LC15	#34
34	A	Tm5	B	5A/B	Straight, small protrusion in a shallower layer	LT11, LC25, LC11, LC15	#33
35	A	TmY11	A	4–6	Perpendicular branches along the short axis	Li16, Li12, LC15, LC10, Li19	
36	A	TmY5	C	4–6	Many short protrusions	LC20, Li12, LC22, LC10, Li19	
37	C	mixture	NA	4–6		LC10, LC20	#32–36
38	B	Tm4 or TmY2 and TmY7	B	1–5A	Short, vertical, Y-shaped ending + knobby	LC4, Li17, mALC1, LPLC1, LC12	#6
39	A	?	NA	4–6	Branching, knobby endings	Li14, LC10, PS179, LPLC2, LC14	
40	A	TmY5a	C	3–6	Vertically diffuse, bistratified, minor protrusions in between	LC10, Li11, LPLC2, LT51, LC4	

The homogeneity of each cluster was ranked in three tiers: A, likely single cell type; B, several cell types; and C, mixture of many cell types. Cell types are labeled where identified, and a question mark is used in cases of cell types that appear homogeneous but are previously undocumented. The confidence of cell-type identification was also ranked in three tiers (A, B, and C for highest to lowest confidence) or marked NA for clusters where we were unable to find cell types.

#### Inputs into columnar visual projection neurons

**Figure 5.**
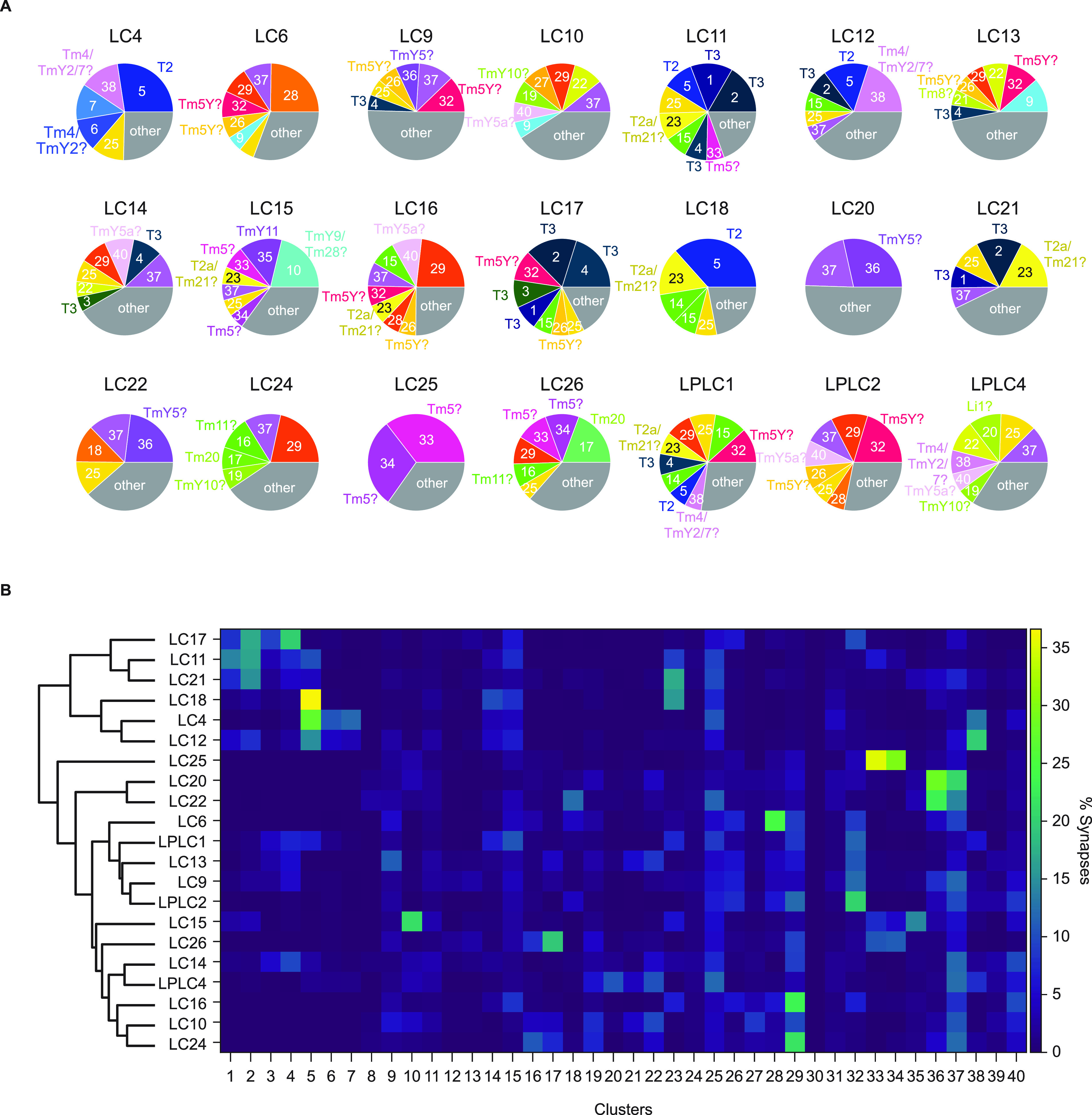
Inputs to columnar visual projection neurons. ***A***, Relative connectivity from the 40 clusters of the cells of interest and LC/LPLC neurons. Clusters with <5% of synaptic input from cells of interest were lumped into “others.” Putative cellular identities of clusters are also shown. Labels with question marks indicate confidence less than B in Table 1. ***B***, Relative connectivity from the 40 clusters to the 21 LC/LPLC types, with a dendrogram from the agglomerative clustering analysis of the LP/LPLC types. Connectivity is normalized for each LC/LPLC type, such that each row of the connectivity matrix adds up to 1.

To gain insight into the visual computations of columnar visual projection neurons in the lobula, we calculated connectivity between the 40 clusters of the cells of interest and 21 LC and LPLC neurons (Wu et al., 2016). Figure 5*A* summarizes major presynaptic clusters for each of the 21 LC/LPLC type, with putative cellular identities of the clusters labeled. Figure 5*B* also shows relative connectivity between 40 clusters and the 21 LC/LPLC neurons, but with a dendrogram from the agglomerative clustering analysis. The most fundamental divide in the dendrogram was between “shallow” LCs with prominent Lo2/3 innervation (LC4, 11, 12,17, 18, 21; Fig. 2*A*,*B*) and the rest. Among the six shallow LC types, LC11, 17, and 21 received more inputs from T3 (clusters 1–4), whereas LC4, 12, and 18 received more inputs from T2 (cluster 5). Among the remaining cell types, LC25 stood out for its almost exclusive connectivity to Tm5 neurons. LC20 and LC22 also had a relatively distinct connectivity pattern, with prominent connection to cluster 36 (putative TmY5). The remaining cell types were divided into two branches, one of which included LC6, 9, 13 and LPLC1, 2, and the other including LC10, 14, 15, 16, 24, 26, and LPLC4. The former branch stood out for their shared connectivity to clusters 26 and 32, putative Tm5Y. The clusters shared by the latter branch included clusters 19 (putative TmY10) and 40 (putative TmY5a). Additionally, we found some clusters that provide substantial inputs to only single LC/LPLC type analyzed here. For example, LC15 was the only cell type that received >5% of its inputs from cluster 10 (putative TmY9/Tm28). Similarly, LC6 was the unique target of cluster 28 and LC22 was the unique target of cluster 18. We could not find morphologic matches for neither cluster 18 or 28, but they were morphologically homogeneous, likely representing genuine cell types.

### The analyses of individual clusters

The following describes the detailed morphologic analyses of the 40 clusters of the cells of interest resulting from the agglomerative clustering (Fig. 3), on which putative cell type identification in Table 1 and Figures 4, 5 are based.

#### Clusters 1 through 4

**Figure 6.**
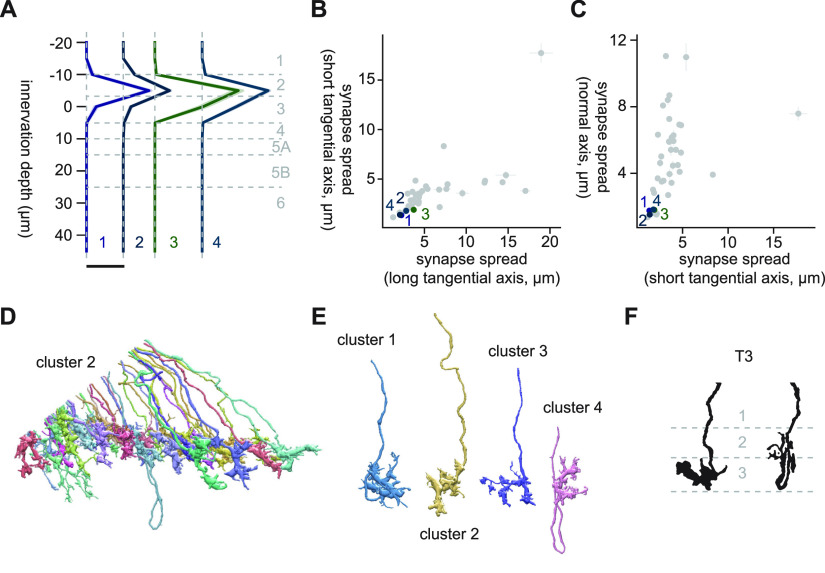
Morphology of clusters 1 through 4, likely corresponding to T3. ***A***, Mean innervation depth and (***B***, ***C***) synapse spread along the three axes of clusters 1 through 4. Error bars indicate SEM. ***A***, Vertical dotted line indicates zero synapses. The thick horizontal bar indicates 20 synapses. ***D***, A population of cluster 2 cells, viewed from a tangential direction. ***E***, Representative examples of cells in clusters 1 through 4. ***F***, Morphology of T3 axon terminals, from Fischbach and Dittrich (1989).

Clusters 1 through 4 were highly distinct from the other clusters, as can be seen from the dendrogram (Fig. 3*A*) and the UMAP embedding (Fig. 3*E*). Cells in these clusters had small monostratified axon terminals slightly elongated along the long tangential axis (synapse spread of ∼2.5 μm along the long axis, and 1.5 μm along the other axes), which innervated the boundary between layers 2 and three of the lobula (i. e., Lo2 and Lo3; Fig. 6*A–C*). The major synaptic targets of these clusters were LC17 (13.7, 6.4, 25.0, and 9.9 synapses/cell for clusters 1, 2, 3, and 4, respectively), LC11 (15.8, 4.2, 6.3, and 2.5 synapses/cell for clusters 1, 2, 3, and 4, respectively), LT1 (8.1, 3.1, 22.4, and 12.3 synapses/cell for clusters 1, 2, 3, and 4, respectively). LPLC1 was also a major target for clusters 3 and 4 (8.7 and 2.5 synapses/cell for clusters 3 and 4, respectively). Visually, the four clusters appeared homogeneous, consisting of cells with small crumpled terminals (Fig. 6*D*,*E*). Their main neurites sometimes overshot and then came back to the layers 2 and 3, forming characteristic loops. Based on their extensive connectivity to LC11 (Keleş et al., 2020; Tanaka and Clark, 2020) and distinctive morphology (Fischbach and Dittrich, 1989; Fig. 6*E*,*F*), these clusters most likely correspond to T3. T3 is a cholinergic (Konstantinides et al., 2018) neuron that connects the proximal medulla (M9) and distal lobula, is an ON-OFF cell, and is tightly tuned to small moving objects (Keleş et al., 2020; Tanaka and Clark, 2020). T3 neurons receive cholinergic inputs from both ON (Mi1) and OFF (Tm1) cells (Takemura et al., 2015), which likely implement their ON-OFF property.

#### Clusters 5 through 7

**Figure 7.**
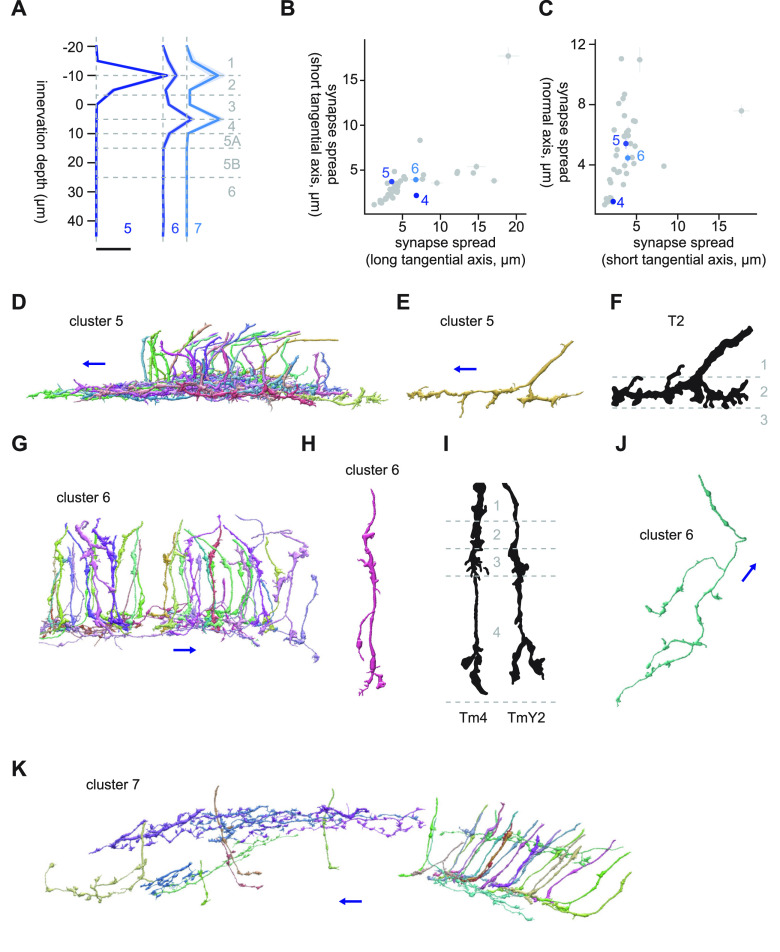
Morphology of clusters 5 through 7. ***A***, Mean innervation depth and (***B***, ***C***) synapse spread along the three axes of clusters 5 through 7. Error bars indicate SEM. ***A***, Vertical dotted line indicates zero synapses. The thick horizontal bar indicates 20 synapses. ***D***, A population of cluster 5 cells, viewed from a tangential direction. ***E***, A representative cluster 5 cell. ***F***, Morphology of T2 axon terminals, from Fischbach and Dittrich (1989). ***G***, A population of cluster 6 cells, viewed from a tangential direction. ***H***, A typical “vertical” cell in cluster 6. ***I***, Morphology of Tm4 and TmY2, from Fischbach and Dittrich (1989). ***J***, A typical “monostratified” cell in cluster 6, viewed from a normal direction. ***K***, A population of cluster 7 cells, viewed from a tangential direction. The blue arrows indicate the ventral direction throughout.

The branch containing clusters 5 through 15 was characterized by high synapse spread along the long tangential axis, reflecting their elongated morphology (Fig. 7*A*). Clusters 5 through 7 had LC4, a loom sensitive visual projection neuron (von Reyn et al., 2014; Ache et al., 2019), as their main postsynaptic target (11.5, 16.9, and 31.6 synapses/cell for clusters 5, 6, and 7, respectively). Cluster 5 was a homogeneous cluster mostly consisting of monostratified cells with T-shaped, spiny axon terminals extending along the long tangential axis, which innervated slightly shallower than the clusters 1 through 4 (T3; Fig. 7*A–E*). Its other major targets included LC11 (3.8 synapses/cell), LC18 (3.5 synapses/cell), Li15 (2.6 synapses/cell), and LPLC1 (2.5 synapses/cell). Based on their morphology (Fischbach and Dittrich, 1989; Fig. 7*E*,*F*) and connectivity to LC11 (Keleş et al., 2020), cluster 5 most likely represents T2 neurons. T2 neurons, similar to T3 neurons, are cholinergic (Konstantinides et al., 2018) ON-OFF cells with tight size tuning (Keleş et al., 2020; Tanaka and Clark, 2020). T2 neurons, again similar to T3 neurons, have bushy dendrites in medulla layer 9, but also have additional dendrites in the proximal medulla (Fischbach and Dittrich, 1989). Their known inputs include both excitatory ON and OFF cells (Mi1, Tm2, L4, and L5; Takemura et al., 2015).

Clusters 6 and 7 were somewhat heterogeneous clusters with small membership (Fig. 7*G*). They were slightly more vertically diffuse than cluster 5 (Fig. 7*A*,*C*). Cluster 6 consisted of at least two cell types: small vertical cells with Y-shaped ending at Lo4 and swelling at Lo2 (Fig. 7*H*), and thin monostratified cells in Lo4 with long, knobby neurites branching perpendicularly (Fig. 7*J*). Cluster 7 was similar to cluster 6 but contained horizontal spiny cells in Lo2, which appeared to be fragmented parts of larger cells (Fig. 7*K*). Their major targets after LC4 were LPLC1 (2.1 synapses/cell) for cluster 6 and LC12 (2.0 synapses/cell) for cluster 7, but these synapse counts were about an order of magnitude lower than their connectivity to LC4. The terminal morphology of the vertical cells resembled Tm4 (Fischbach and Dittrich, 1989; Fig. 7*I*), although Tm4 appears to have more extensive swelling in Lo1. TmY2 also had similar Y-shaped ending (Fig. 7*I*), but they have swelling in Lo3 rather than Lo2 (Fischbach and Dittrich, 1989).

#### Clusters 8 through 11

**Figure 8.**
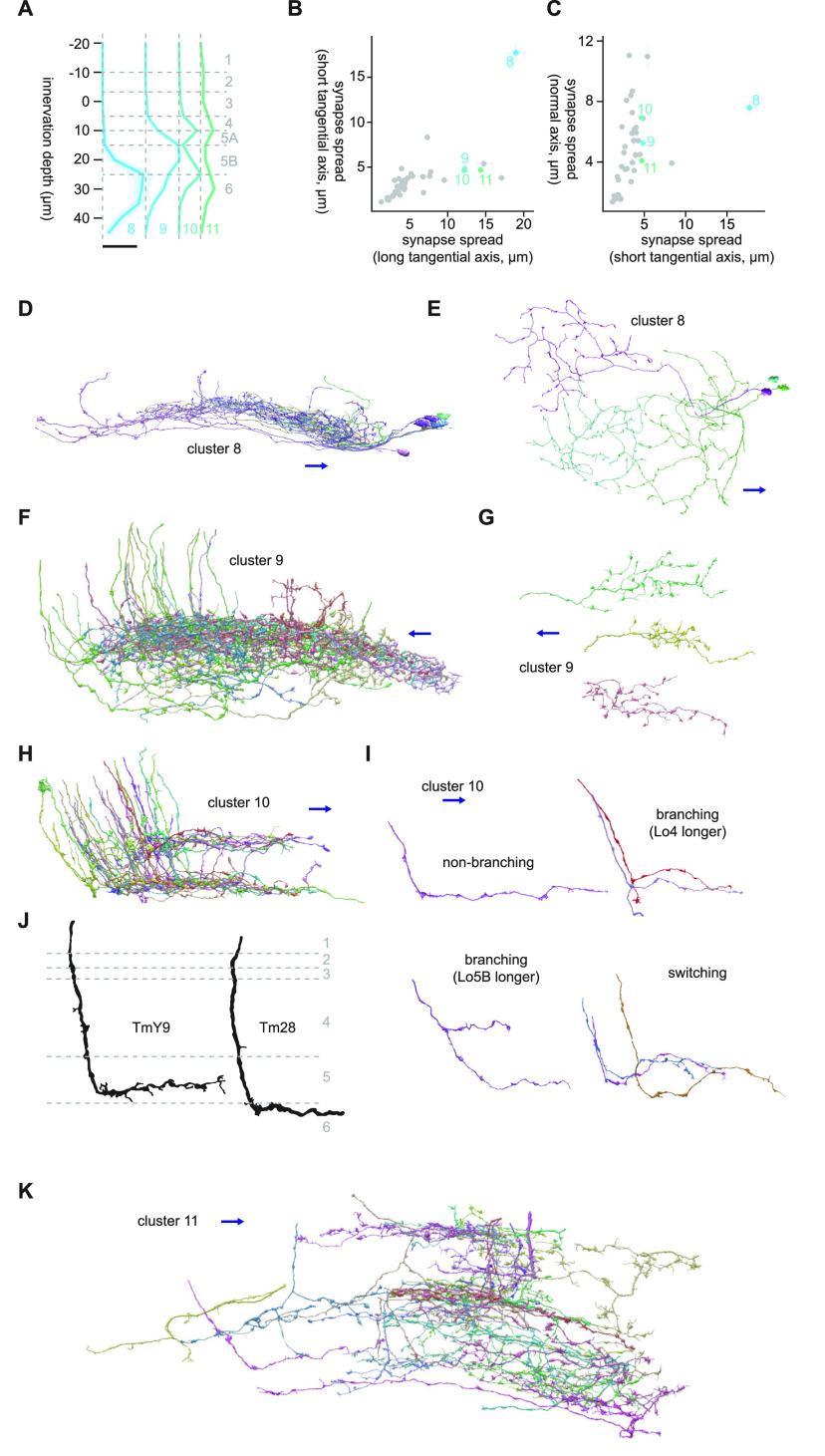
Morphology of clusters 8 through 11. ***A***, Mean innervation depth and (***B***, ***C***) synapse spread along the three axes of clusters 8 through 11. Error bars indicate SEM. ***A***, Vertical dotted line indicates zero synapses. The thick horizontal bar indicates 20 synapses. ***D***, A population of cluster 8 cells, viewed from a tangential direction. ***E***, Representative cluster 8 cells, viewed from a normal direction. ***F***, A population of cluster 9 cells, viewed from a tangential direction. ***G***, Representative cluster 9 cells, viewed from a normal direction. ***H***, A population of cluster 10 cells, viewed from a tangential direction. ***I***, Representative cluster 10 cells, viewed from a normal direction. Four different subtypes are shown, as labeled. ***J***, Morphology of TmY9 and Tm28 axon terminals, from Fischbach and Dittrich (1989). ***K***, A population of cluster 11 cells, viewed from a tangential direction. The blue arrows indicate the ventral direction throughout.

Clusters 8 through 11 were characterized by their large synapse spread along the long tangential axis, as well as the normal axis (Fig. 8*B*,*C*). They generally innervated deeper than Lo4 (Fig. 8*A*), and shared connectivity to LC10 (7.6, 9.0, 3.0, and 1.4 synapses/cell for clusters 8, 9, 10, and 11, respectively), a group of projection neurons involved in male courtship rituals (Ribeiro et al., 2018; Sten et al., 2021). Cluster 8 consisted of large, tangentially isotropic neurons monostratified in Lo6, likely representing an as yet unlabeled lobula intrinsic (Li) neuron type (Fig. 8*D*,*E*). Their somata sat on the ventral end of the lobula, and their main neurite entered proximally into the lobula (Fig. 8*D*). Each of these cells covered about a quarter of the tangential extent of the lobula (Fig. 8*E*). Their major postsynaptic targets after LC10 were LC20 (5.9 synapses/cell), mALC2 (5.2 synapses/cell), LT52 (5.2 synapses/cell), and Li12 (4.4 synapses/cell).

Cluster 9 was a highly anisotropic cluster with synapse spread of 12.2 and 4.8 μm along short and long tangential axes (Fig. 8*B*). The dominant cell type of this cluster had a long, knobby neurites branching at acute angles in Lo5B, which entered the lobula ventrally and then bent dorsally (Fig. 8*F*,*G*). The major postsynaptic targets of this cluster after LC10 were LC13 (5.1 synapses/cell), Li19 (3.8 synapses/cell), LC6 (2.3 synapses/cell), and LT57 (2.0 synapses/cell).

Cluster 10 was another highly anisotropic cluster with synapse spread of 12.2 and 4.6 μm along short and long tangential axes (Fig. 8*B*). The major postsynaptic targets of cluster 10 other than LC10 were Li16 (5.5 synapses/cell), Li12 (4.5 synapses/cell), LC15 (2.7 synapses/cell), and Li13 (2.4 synapses/cell). The dominant cell type of this cluster had a linear neurite with little branching that entered the lobula dorsally and then bent ventrally almost perpendicularly (Fig. 8*H*). While this cluster appears to be bistratified in Lo4 and Lo5B as a population (Fig. 8*H*), closer inspection revealed variations in the morphology of single cells (Fig. 8*I*): some cells were genuinely bistratified, but the branches were often longer in one layer than the other. Some other cells entirely lacked branching. Interestingly, some of these non-branching cells initially entered Lo5B and then switched to Lo4. This long, linear terminal morphology resembled TmY9 (Fischbach and Dittrich, 1989; Fig. 8*J*). TmY9 is known to have long, linear neurites similar to its lobula terminal in the medulla and lobula plate as well (Fischbach and Dittrich, 1989). Another known cell type with long, linear terminals oriented ventrally is Tm28 (Fischbach and Dittrich, 1989; Fig. 8*J*), although they appear to innervate a little deeper. Note that, although the drawing of TmY9 in Fischbach and Dittrich (1989) lacks branching, *Musca* Y17 cells, a putative TmY9 homolog, has a bistratified branching (Douglass and Strausfeld, 1998), similar to what is shown here.

Cluster 11 was a highly heterogeneous cluster with a variety of cell types in deeper layers of the lobula with elongated morphology along the long tangential axis (Fig. 8*K*). It appeared to contain cells similar to those in clusters through 8–10. The largest postsynaptic target of this cluster was LC10, but the mean synapse count per cell was only 1.4, likely reflecting the heterogeneity of the cluster.

#### Clusters 12 through 15

**Figure 9.**
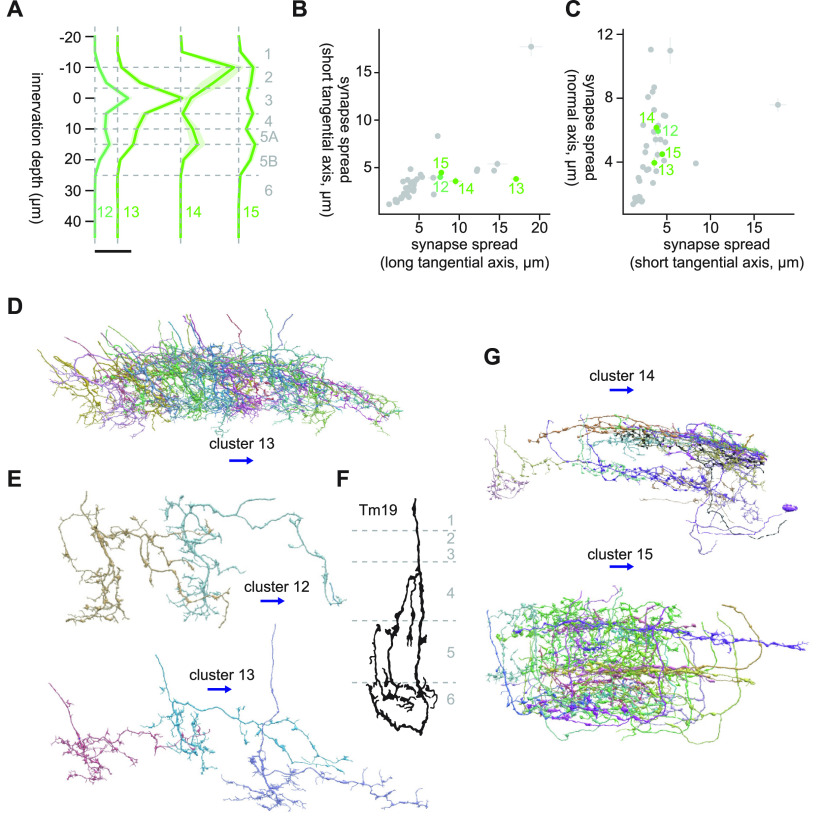
Morphology of clusters 12 through 15. ***A***, Mean innervation depth and (***B***, ***C***) synapse spread along the three axes of clusters 12 through 15. Error bars indicate SEM. ***A***, Vertical dotted line indicates zero synapses. The thick horizontal bar indicates 20 synapses. ***D***, A population of cluster 13 cells, viewed from a tangential direction. ***E***, Representative cluster 12 and 13 cells, viewed from a normal direction. ***F***, Morphology of Tm19 axon terminals, from Fischbach and Dittrich (1989). ***G***, A population of cluster 14 and 15 cells, viewed from a tangential direction. The blue arrows indicate the ventral direction throughout.

Clusters 12 through 15 are another set of clusters with large synapse spread along the long tangential axis (Figs. 3*A*, 9*B*,*C*). These clusters innervated shallower, around Lo2 to Lo4 (Fig. 9*A*), unlike the previous four clusters. Clusters 12 and 13 were homogeneous clusters dominated by vertically diffuse cells that shared connection to LT87 (10.9 and 19.0 synapses/cell for clusters 12 and 13, respectively), as well as to LC31 (2.9 and 12.2 synapses/cell for clusters 12 and 13, respectively) and LC17 (2.4 and 9.9 synapses/cell for clusters 12 and 13, respectively; Fig. 9*D*). These cells had a meandering main neurite with many minor branches (Fig. 9*E*). In addition, they had a long secondary branch sticking out ventrally from the main neurite perpendicularly at around Lo3, which was also meandering and with many minor branches (Fig. 9*E*). Among previously documented cells, Tm19 appeared similar to these clusters in that it has an extensive secondary branch (Fischbach and Dittrich, 1989; Fig. 9*F*), but their innervation appears deeper.

Unlike clusters 12 and 13, clusters 14 and 15 were highly heterogeneous and it was difficult to make out the dominant cell types (Fig. 9*G*). The main postsynaptic targets of these clusters were LPLC1 (19.9 and 5.7 synapses/cell for clusters 14 and 15, respectively). Cluster 14 contained some T2 cells, as can be also seen from the UMAP embedding, where some cluster 14 cells are next to the T2-dominated cluster 5.

#### Clusters 16 and 17

**Figure 10.**
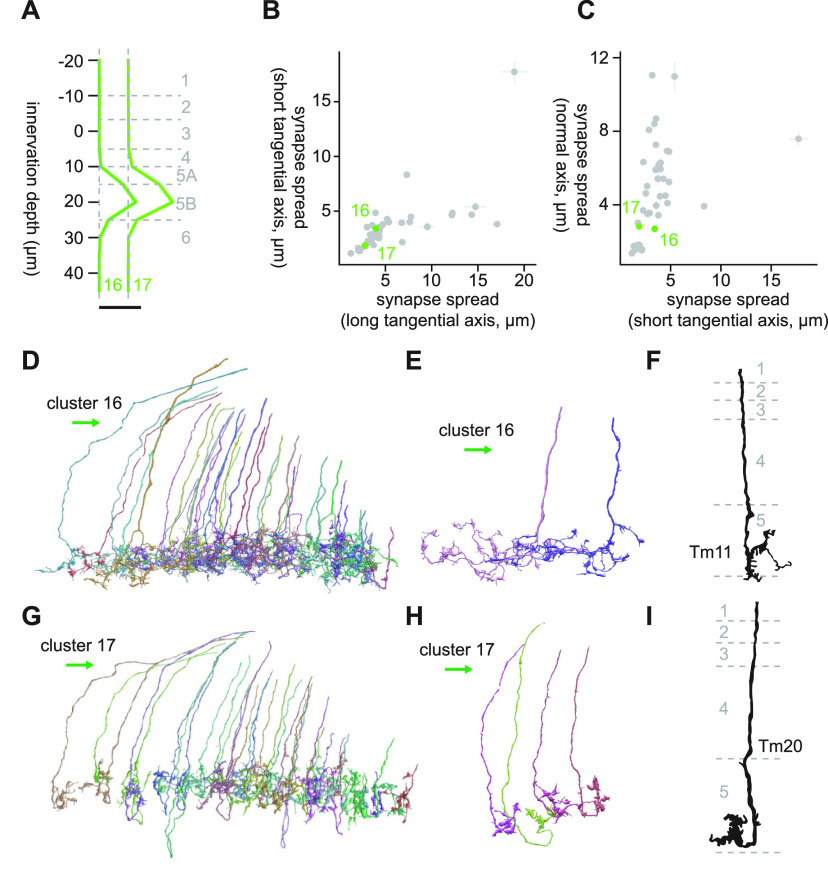
Morphology of clusters 16 and 17. ***A***, Mean innervation depth and (***B***, ***C***) synapse spread along the three axes of clusters 16 and 17. Error bars indicate SEM. ***A***, Vertical dotted line indicates zero synapses. The thick horizontal bar indicates 20 synapses. ***D***, A population of cluster 16 cells, viewed from a tangential direction. ***E***, Representative cluster 16 cells. ***F***, Morphology of Tm11 axon terminals, from Fischbach and Dittrich (1989). ***G***, A population of cluster 17 cells, viewed from a tangential direction. ***H***, Representative cluster 17 cells. ***I***, Morphology of a Tm20 axon terminal, from Fischbach and Dittrich (1989). The green arrows indicate the anterior direction throughout.

The branch containing clusters 16 through 22 were characterized by their innervation being relatively restricted to the deep layers, Lo5B and Lo6 (Fig. 10*A*). Clusters 16 and 17 were monostratified clusters in Lo5B with small synapse spread around 3 μm in every direction (Fig. 10*B*,*C*). They also shared their connectivity to LT58 (8.4 and 12.8 synapses/cell for clusters 16 and 17, respectively), LC10 (4.3 and 4.2 synapses/cell for clusters 16 and 17, respectively), and LC24 (2.6 and 1.8 synapses/cell for clusters 16 and 17, respectively). However, the two clusters appeared to have distinct terminal morphology. Cells in cluster 16 had more tangentially extensive terminals, which consisted of thin, knobby neurites branching perpendicularly, oriented posteriorly (Fig. 10*D*,*E*). In contrast, the terminals of cluster 17 cells were thicker and branched less (Fig. 10*G*,*H*). The main neurite of these cells appeared to touch the bottom of Lo5B first and then curl upward, creating a distinctive looped morphology similar to those of T3 (Fig. 10*H*). In terms of connectivity, clusters 17 differed from cluster 16 in that it had a large number of synapses on mALC2 (9.6 synapses/cell). The morphology of cluster 17 cells highly resembled that of Tm20 (Fischbach and Dittrich, 1989; Fig. 10*I*). The closest match we could find for cluster 16 was Tm11 (Fig. 10*F*), although we are not particularly confident about this identification (Fischbach and Dittrich, 1989). Tm20 is a cholinergic cell (Davis et al., 2020) that is directly postsynaptic to spectrally sensitive photoreceptor R8 (Gao et al., 2008; Takemura et al., 2015), necessary for color learning combinedly with Tm5 subtypes (Melnattur et al., 2014). Other major inputs to Tm20 include L2, L3, C3, Tm1, and Mi4 (Takemura et al., 2015). LT11, a tangential projection neuron necessary for wavelength-specific phototaxis (Otsuna et al., 2014), was not among the strongest postsynaptic target of cluster 17, although LT11 was previously suggested to be downstream of Tm20 based on transsynaptic GFP reconstruction (GRASP; Lin et al., 2016).

#### Clusters 18 and 19

**Figure 11.**
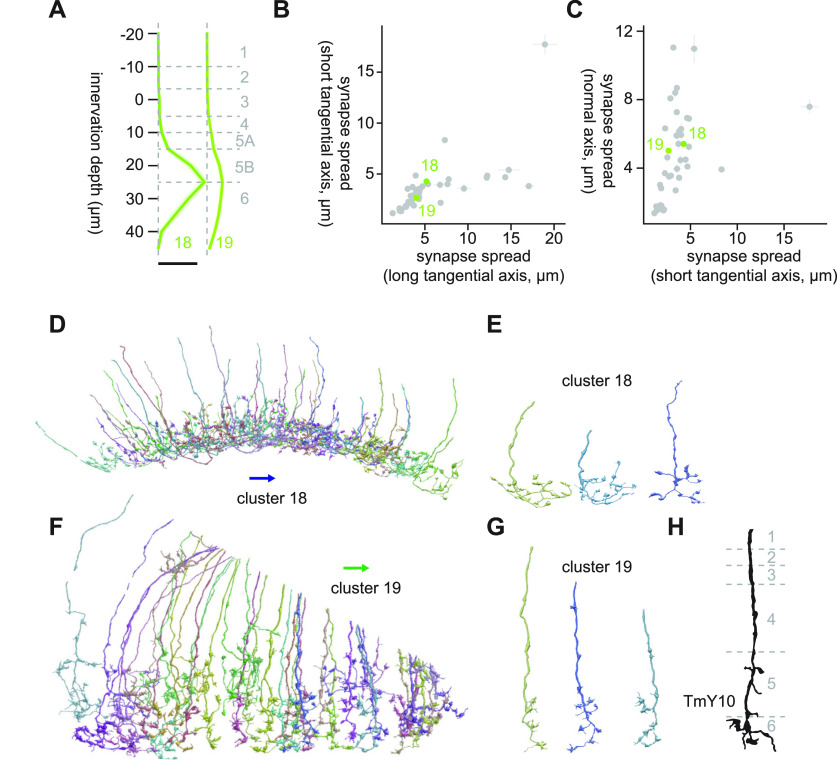
Morphology of clusters 18 and 19. ***A***, Mean innervation depth and (***B***, ***C***) synapse spread along the three axes of clusters 18 and 19. Error bars indicate SEM. ***A***, Vertical dotted line indicates zero synapses. The thick horizontal bar indicates 20 synapses. ***D***, A population of cluster 18 cells, viewed from a tangential direction. ***E***, Representative cluster 18 cells. ***F***, A population of cluster 19 cells, viewed from a tangential direction. ***G***, Representative cluster 19 cells. ***H***, Morphology of a TmY10 axon terminal, from Fischbach and Dittrich (1989). The blue and green arrows, respectively, indicate the ventral and anterior directions.

Clusters 18 and 19 were deep monostratified clusters similar to clusters 16 and 17, but they innervated slightly deeper, reaching Lo6 (Fig. 11*A*). They also shared connectivity to mALC2 (14.7 and 7.5 synapses/cell for clusters 18 and 19, respectively), but their postsynaptic targets were distinct beyond that: cluster 18 synapsed onto LPLC2 (4.8 synapses/cell), LT58 (4.1 synapses/cell), LC22 (3.8 synapses/cell), and LC6 (3.6 synapses/cell), while cluster 19 synapsed onto LC10 (3.6 synapses/cell), mALC1 (2.4 synapses/cell), and Li12 (1.3 synapses/cell). Cluster 18 consisted of homogeneous cells whose terminals had short branches with knobby endings (Fig. 11*D*,*E*). The dominant cell type of cluster 19 had terminals with fewer branches compared with cluster 18, but they were vertically more diffuse and had small protrusions in shallower layers (Fig. 11*F*,*G*). The best morphologic matches we could find for these clusters were TmY10 (Fischbach and Dittrich, 1989; Fig. 11*H*), but we are not particularly confident with this identification.

#### Clusters 20 through 22

**Figure 12.**
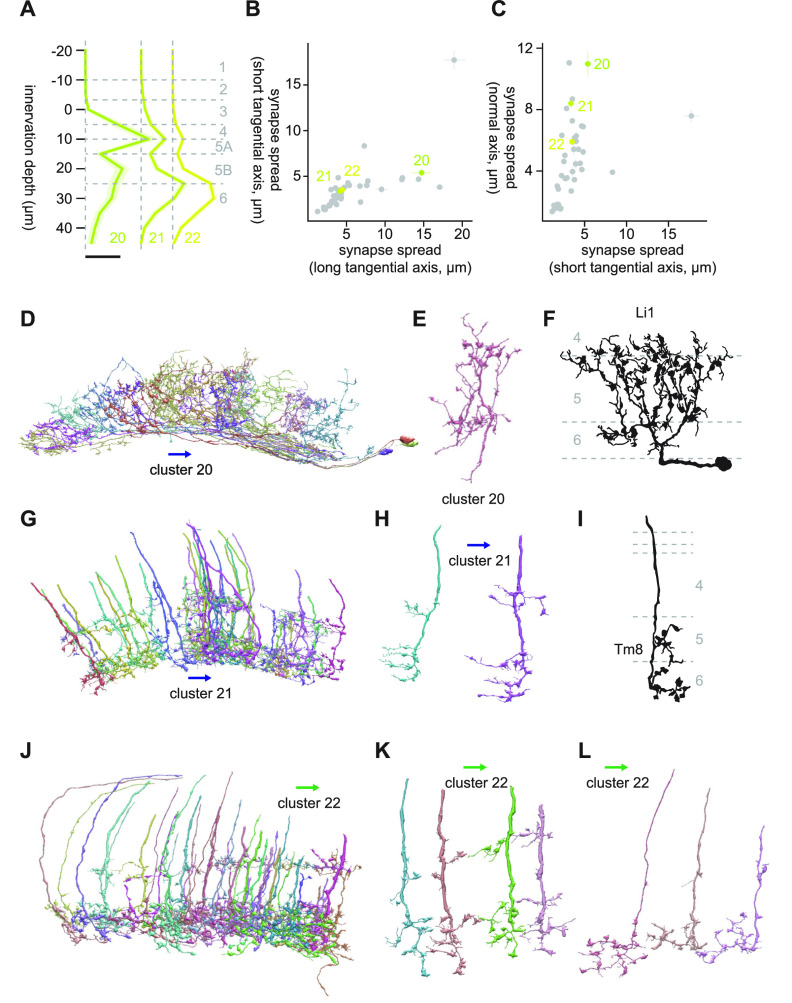
Morphology of clusters 20 through 22. ***A***, Mean innervation depth and (***B***, ***C***) synapse spread along the three axes of clusters 20 through 22. Error bars indicate SEM. ***A***, Vertical dotted line indicates zero synapses. The thick horizontal bar indicates 20 synapses. ***D***, A population of cluster 20 cells, viewed from a tangential direction. ***E***, A representative cluster 20 cell. ***F***, Morphology of a Li1 cell, from Fischbach and Dittrich (1989). ***G***, A population of cluster 21 cells, viewed from a tangential direction. ***H***, Representative cluster 21 cells. ***I***, Morphology of a Tm8 axon terminal, from Fischbach and Dittrich (1989). ***J***, A population of cluster 22 cells, viewed from a tangential direction. ***K***, ***L***, Representative (***K***) thick and (***L***) thin cells in cluster 22. The blue and green arrows, respectively, indicate the ventral and anterior directions.

Clusters 20 through 22 share their strong connectivity to mALC2 with the previous two clusters (7.5, 24.3, and 19.7 synapses/cell for clusters 20, 21, and 22, respectively). Unlike clusters 16 through 19, they were bistratified (Fig. 12*A*). Cluster 20 consisted of large, lobula intrinsic (Li) neurons bistratified in Lo6 and Lo4/5A, whose cell bodies were on the ventral end of the lobula (Fig. 12*D*). Their bistratified morphology, as well as knobby appearance (Fig. 12*D*,*E*), resembled Li1 (Fischbach and Dittrich, 1989; Fig. 12*F*). Major postsynaptic targets of this cluster other than mALC2 were LPLC4 (10.7 synapses/cell), LC10 (6.7 synapses/cell), Li13 (5.7 synapses/cell), and LT36 (4.9 synapses/cell).

Cluster 21 consisted of cells with thick neurite, which are bistratified in Lo6 and Lo5A (Fig. 12*A*,*G*,*H*). Individual neurons in this cluster had a single side branch in Lo5A and multiple branches in Lo6, all oriented dorsally (Fig. 12*H*). These oriented branches resembled those of Tm8 (Fischbach and Dittrich, 1989; Fig. 12*I*). The major postsynaptic targets of this cluster after mALC2 were LC10 (15.5 synapses/cell), LT57 (5.1 synapses/cell), LC13 (4.9 synapses/cell), and LT52 (3.7 synapses/cell). Cluster 22 consisted of at least two distinct cell types (Fig. 12*J–L*). One was thick, bistratified cells similar to cluster 21 (Fig. 12*K*), but their branches were not oriented like cluster 21 cells. The other was thin cells that had terminals with branches with knobby endings and a small protrusion at a shallower layer (Fig. 12*L*). Their major postsynaptic targets after mALC2 were LC10 (8.2 synapses/cell), LT52 (4.1 synapses/cell), LT63 (2.6 synapses/cell), and LT51 (2.6 synapses/cell).

#### Cluster 23

**Figure 13.**
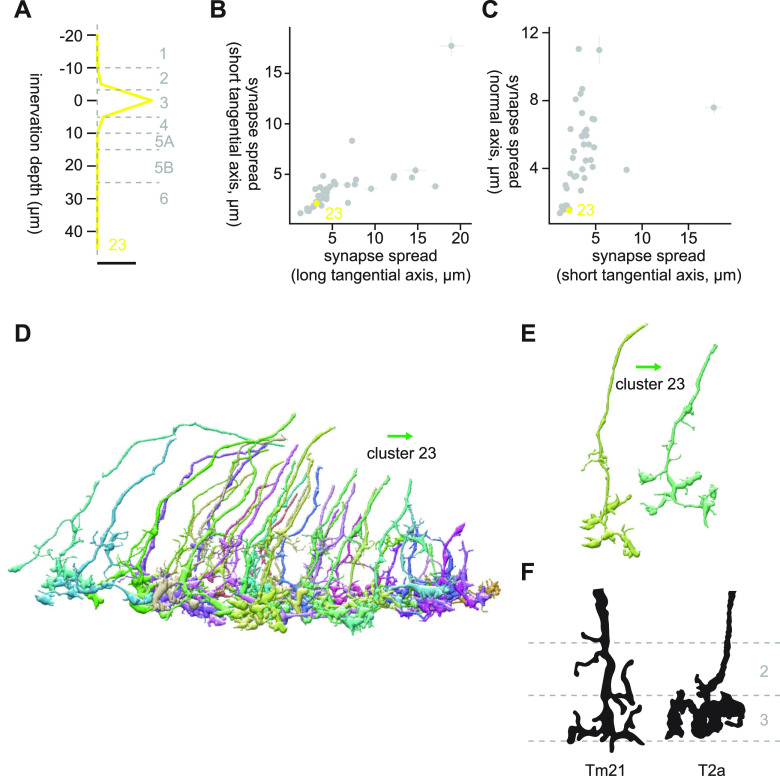
Morphology of cluster 23. ***A***, Mean innervation depth and (***B***, ***C***) synapse spread along the three axes of cluster 23. Error bars indicate SEM. ***A***, Vertical dotted line indicates zero synapses. The thick horizontal bar indicates 20 synapses. ***D***, A population of cluster 23 cells, viewed from a tangential direction. ***E***, Representative cluster 23 cells. ***F***, Morphology of Tm21 and T2a cells, from Fischbach and Dittrich (1989). The green arrows indicate the anterior direction.

Cluster 23 was highly distinct from the neighboring clusters, as can be seen both from the dendrogram (Fig. 3*A*) and UMAP embedding (Fig. 3*E*). Cluster 23 consists of a small monostratified cells in Lo3, with synapse spread of around 2 μm in every direction (Fig. 13*A–C*). Its main postsynaptic targets were Li11 (8.5 synapses/cell), LT1 (4.8 synapses/cell), LC11 (2.6 synapses/cell), LPLC1 (2.1 synapses/cell), and LC21 (1.8 synapses/cell). Visually, cluster 23 appeared to consist of highly homogenous cells with a knobby terminal and a small protrusion at a shallower location (Fig. 13*D*,*E*). The best morphologic match we could find was T2a, a cell type morphologically and transcriptomically related to T2 and T3 (Fischbach and Dittrich, 1989; Keleş et al., 2020; Özel et al., 2021; Fig. 13*F*). Indeed, this cluster shared its connectivity to LT1, LC11, and LPLC1 with T3, indicating that it could be functionally similar to T3. While functional properties of T2a have not been studied, it shares inputs from Mi1 and Tm1 with T3, implying it is also an ON-OFF cell (Takemura et al., 2015). T2a lacks inputs from L cells unlike T2, despite its proximal medulla innervation (Takemura et al., 2015). Another cell type with similar terminal morphology was Tm21 (Fischbach and Dittrich, 1989; Fig. 13*F*).

#### Clusters 24 and 25

**Figure 14.**
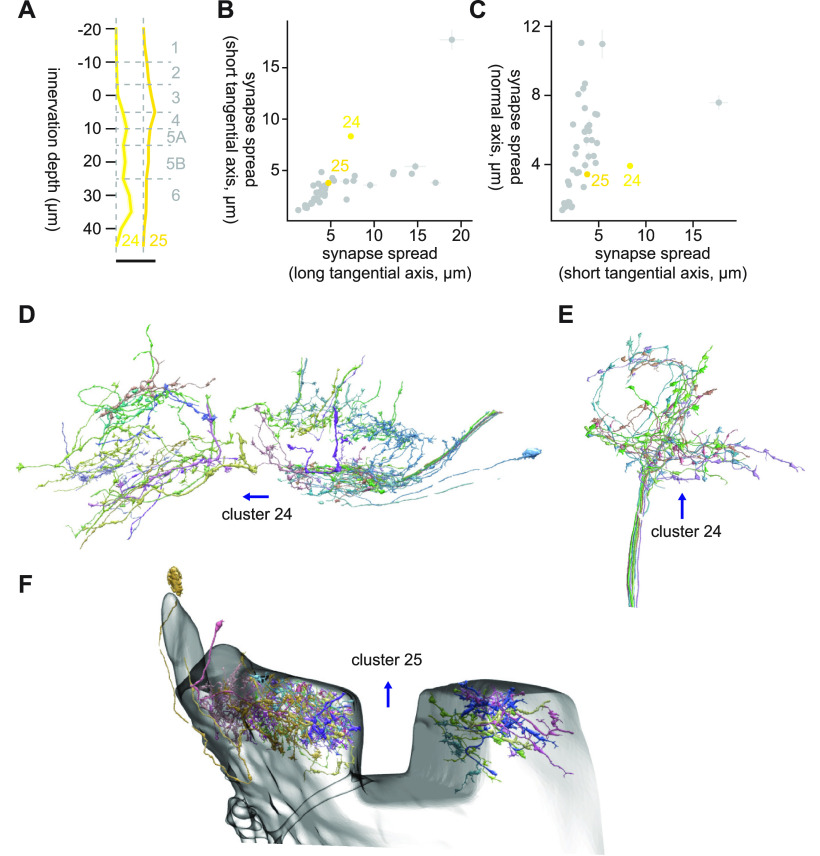
Morphology of clusters 24 and 25. ***A***, Mean innervation depth and (***B***, ***C***) synapse spread along the three axes of clusters 24 and 25. Error bars indicate SEM. ***A***, Vertical dotted line indicates zero synapses. The thick horizontal bar indicates 20 synapses. ***D***, A population of cluster 24 cells, viewed from a tangential direction. ***E***, Cluster 24 cells whose process entered the lobula from proximal and dorsal end. ***F***, A population of cluster 25 cells around the dent in the lobula. The transparent shell represents the outer boundary of the lobula. The blue arrows indicate the ventral direction. Clusters 26 through 29.

Clusters 24 and 25 were highly heterogeneous clusters, and it was difficult to make out the dominant cell types (Fig. 14*D*,*F*). The low synapse counts to even their largest targets (cluster 24: 2.16 synapses/cell to mALC2; cluster 25: 1.2 synapses/cell to LC4) and the very diffuse innervation depth profile (Fig. 12*A*) likely reflect this heterogeneity. Of note, cluster 24 contained a set of neurites that entered the lobula from the dorsal proximal end as if hugging the bottom of Lo6 (Fig. 14*D*,*E*). Cluster 25 contained a large number of fragmented neurites around the dent in the lobula (Fig. 14*F*) which was likely caused by how it was mounted for scanning. Thus, this cluster may be driven by the imaging methodology rather than by the biology.

#### Clusters 26 and 29

**Figure 15.**
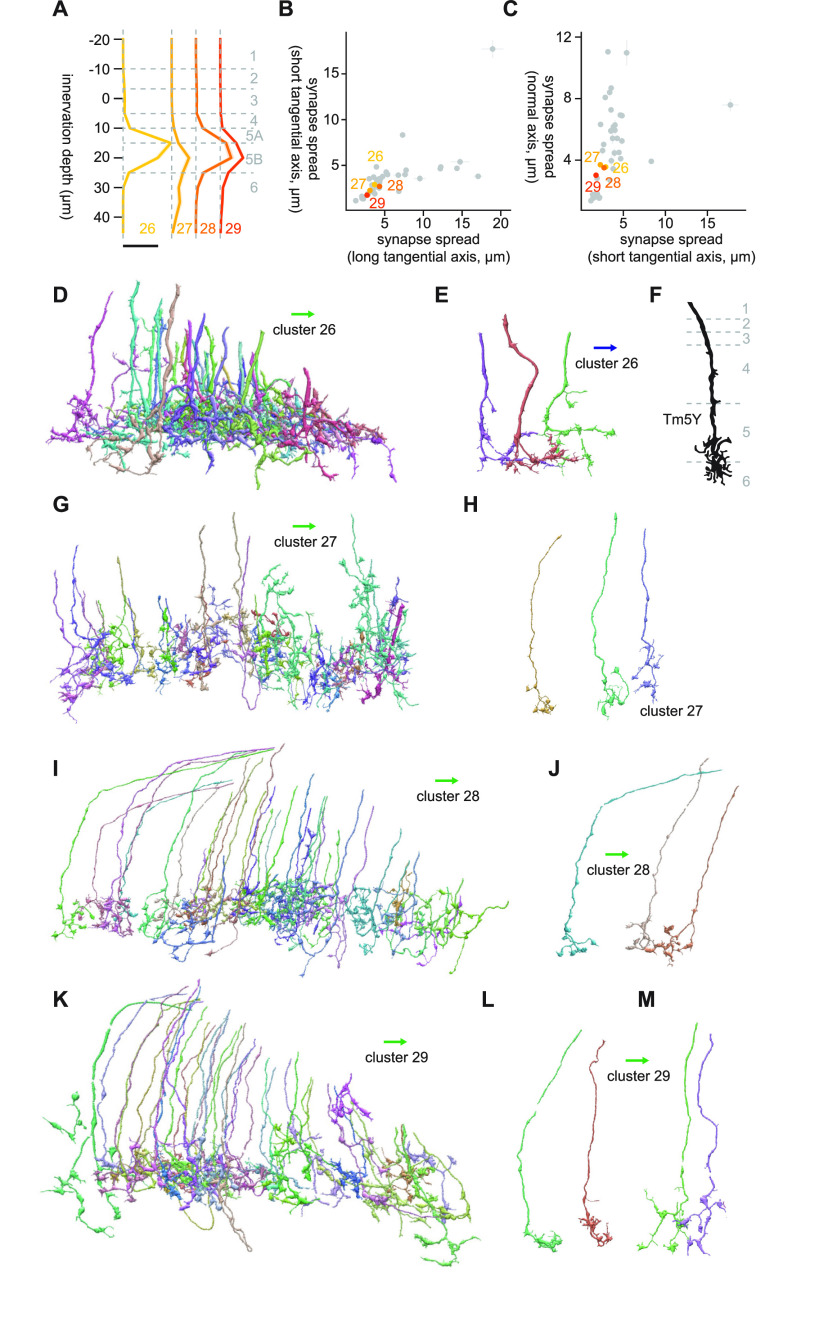
Morphology of clusters 26 through 29. ***A***, Mean innervation depth and (***B***, ***C***) synapse spread along the three axes of clusters 26 through 29. Error bars indicate SEM. ***A***, Vertical dotted line indicates zero synapses. The thick horizontal bar indicates 20 synapses. ***D***, A population of cluster 26 cells, viewed from a tangential direction. ***E***, A representative cluster 26 cell. (***F***) Morphology of a Tm5Y cell, from Fischbach and Dittrich (1989). ***G***, A population of cluster 27 cells, viewed from a tangential direction. ***H***, Representative cluster 27 cells. ***I***, A population of cluster 28 cells, viewed from a tangential direction. ***J***, Representative cluster 28 cells. ***K***, A population of cluster 29 cells, viewed from a tangential direction. ***L***, ***M***, Representative examples of two cell types included in cluster 29. ***L***, These cells were similar to cluster 16 cells (Tm20). The blue and green arrows, respectively, indicate the ventral and anterior directions.

Clusters 26 through 29 were deep monostratified clusters in Lo5A/B (Fig. 15*A*), similar to clusters 16 through 19 (Figs. 6, 10). There terminals were relatively compact, with synapse spread around 3 μm in every direction (Fig. 15*B*,*C*). The major postsynaptic targets of cluster 26 were Li19 (7.2 synapses/cell), LC17 (4.4 synapses/cell), LT79 (2.7 synapses/cell), LPLC2 (2.5 synapses/cell), and LC6 (1.6 synapses/cell). The dominant cell type of this cluster had thick neurites with small protrusions and terminals oriented ventrally with a couple of branches (Fig. 15*D*,*E*). These morphologic features resembled those of Tm5Y (Fischbach and Dittrich, 1989; Fig. 15*F*).

Cluster 27 was somewhat heterogeneous (Fig. 15*G*), and the only major postsynaptic target with more than a single synapse/cell was LC10 (9.6 synapses/cell). The dominant cell type had thin processes and terminals whose short branches had knobby endings (Fig. 15*H*). These features were similar to the thin cells in cluster 22 (Fig. 12*K*), but cluster 27 cells were less tangentially extensive and positioned slightly shallower. Cluster 28 was also dominated by thin, monostratified cells in Lo5B (Fig. 15*I*,*J*), which had knobby endings, similar to cluster 27. However, postsynaptic targets of cluster 28 were distinct from cluster 27: its major targets were LC6 (6.3 synapses/cell), LPLC2 (2.3 synapses/cell), LC10 (2.1 synapses/cell), Li19 (1.7 synapses/cell), and LC16 (1.5 synapses/cell). Cluster 29 was a large, heterogeneous cluster with deep monostratified neurons (Fig. 15*K*). It contained cells similar to cluster 27 (Fig. 15*H*,*M*), as well as to cluster 17 (Tm20; Fig. 15*L*). LC10 (2.1 synapses/cell) and LC16 (1.3 synapses/cell) were among their major targets. We could not find good morphologic match for these monostratified Lo5B cells in Fischbach and Dittrich (1989).

#### Clusters 30 and 31

**Figure 16.**
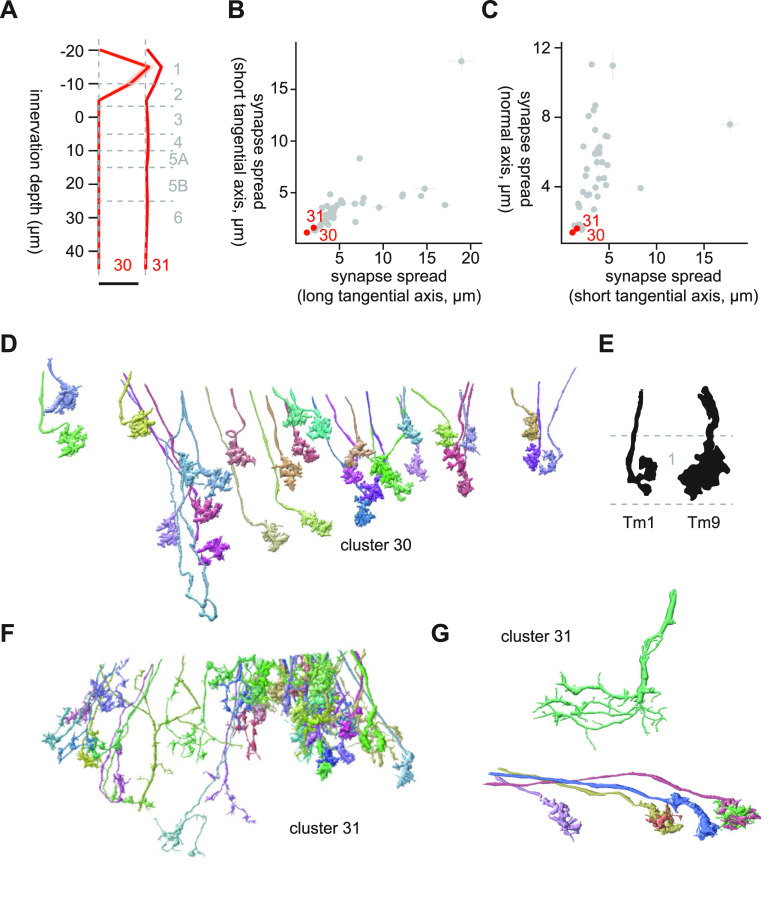
Morphology of clusters 30 and 31. ***A***, Mean innervation depth and (***B***, ***C***) synapse spread along the three axes of clusters 30 and 31. Error bars indicate SEM. ***A***, Vertical dotted line indicates zero synapses. The thick horizontal bar indicates 20 synapses. ***D***, The entire population of cluster 30 cells. ***E***, Morphology of Tm1 and Tm9 axon terminals, from Fischbach and Dittrich (1989). ***F***, A population of cluster 31 cells. ***G***, Examples of cluster 31 cells that resembled T5 (top) and their inputs (bottom).

Clusters 30 and 31 were the only clusters with substantial Lo1 innervation (Fig. 16*A*). Lo1 appears to be a special layer dedicated to OFF edge motion detection, housing T5 and its inputs but avoided by other cell types, including lobula VPNs (Fischbach and Dittrich, 1989; Shinomiya et al., 2014, 2019; Wu et al., 2016). This is similar to how M10, the putative evolutionary precedent of Lo1 (Shinomiya et al., 2015), is dedicated to T4 and their inputs but avoided by other cells (Fischbach and Dittrich, 1989). Cluster 30 was a highly homogeneous cluster of small monostratified terminals (Fig. 16*D*), whose only major postsynaptic target was CT1 (20.9 synapses/cell). CT1 receives inputs from Tm1 as well as Tm9 in the lobula (Shinomiya et al., 2019). The terminal morphology of cluster 30 cells resembled Tm9 more than Tm1 (Fischbach and Dittrich, 1989; Fig. 16*E*).

Cluster 31 was a large heterogeneous cluster mostly confined to Lo1 (Fig. 16*A*,*F*). This cluster appears as multiple distant clusters on the UMAP embedding (Fig. 3*E*), also likely reflecting its heterogeneity. Upon visual inspection, we found oriented neurites with dense branching similar to T5 dendrites, as well as small terminals that resembled Tm1, 2, and 9 (Fig. 16*G*). On average, cluster 31 did not have more than single synapse/cell to any of the labeled lobula neurons included in the current analysis. This observation supports the idea that Lo1 is the dedicated layer for motion detection by T5 and is relatively independent from the rest of the lobula.

#### Cluster 32 through 34

**Figure 17.**
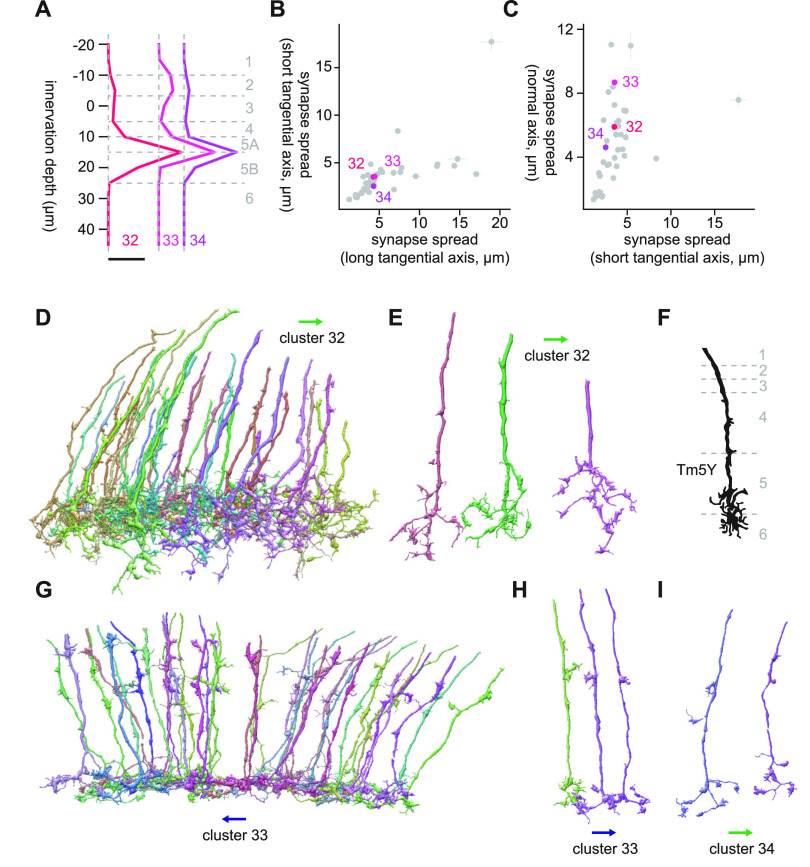
Morphology of clusters 32 through 34. ***A***, Mean innervation depth and (***B***, ***C***) synapse spread along the three axes of clusters 32 through 34. Error bars indicate SEM. ***A***, Vertical dotted line indicates zero synapses. The thick horizontal bar indicates 20 synapses. ***D***, A population of cluster 32 cells. ***E***, Representative examples of cluster 32 cells. ***F***, Morphology of a Tm5Y axon terminal, from Fischbach and Dittrich (1989). ***G***, A population of cluster 32 cells. ***H***, ***I***, Examples of (***H***) cluster 33 and (***I***) cluster 34 cells. The blue and green arrows, respectively, indicate the ventral and anterior directions.

Clusters 32 through 40 were characterized by their tangentially compact and vertically diffuse morphology (Fig. 17*B*,*C*). The axon terminals of clusters 32 through 34 were in Lo5A/B, but they also had synapses in shallower layers (Fig. 17*A*). The major postsynaptic targets of cluster 32 were Li19 (12.9 synapses/cell), LC17 (9.3 synapses/cell), LT79 (8.4 synapses/cell), LPLC2 (8.1 synapses/cell), and LPLC1 (7.4 synapses/cell). These cell types overlapped with the main synaptic targets of cluster 26. In fact, the morphology of cluster 32 cells, with its thick neurites with small protrusions and somewhat densely branching terminals (Fig. 17*D*,*E*), resembled that of cluster 26 cells (Fig. 15*D*,*E*). Continuity of clusters 26 and 32 can also be seen from the UMAP embedding (Fig. 3E). The best morphologic match we could find for these clusters was Tm5Y (Fischbach and Dittrich, 1989; Fig. 17*F*).

Clusters 33 and 34 shared their connectivity to LT11 (20.7 and 11.2 synapses/cell for clusters 33 and 34, respectively), LC25 (7.2 and 4.2 synapses/cell for clusters 33 and 34, respectively), LC11 (5.8 and 1.4 synapses/cell for clusters 33 and 34, respectively), LC15 (2.3 and 1.1 synapses/cell for clusters 33 and 34, respectively), LC26 (1.6 and 1.1 synapses/cell for clusters 33 and 34, respectively). These were the only clusters with strong LT11 connectivity, which most likely made these clusters highly distinct from everything else on the UMAP embedding (Fig. 3*E*). Both clusters were visually homogeneous, consisting of cells characterized by modestly branching terminals in Lo5A/B and straight main neurite with small protrusions in shallower layers (Fig. 17*G–I*). Given their extensive connectivity to LT11, these clusters likely represent Tm5 neurons (Lin et al., 2016). Tm5 neurons, consisting of three subtypes Tm5a/b/c, are glutamatergic neurons postsynaptic to spectrally sensitive photoreceptors R7 and R8 (Gao et al., 2008). Tm5 subtypes, alongside with Tm20, have been shown to be combinedly necessary for color learning in flies (Karuppudurai et al., 2014; Melnattur et al., 2014). Among these subtypes, Tm5c, resembled the cluster 33 and 34 cells the most in that it had a small protrusion in a shallower layer in addition to Lo5 (Gao et al., 2008).

#### Clusters 35 through 37

**Figure 18.**
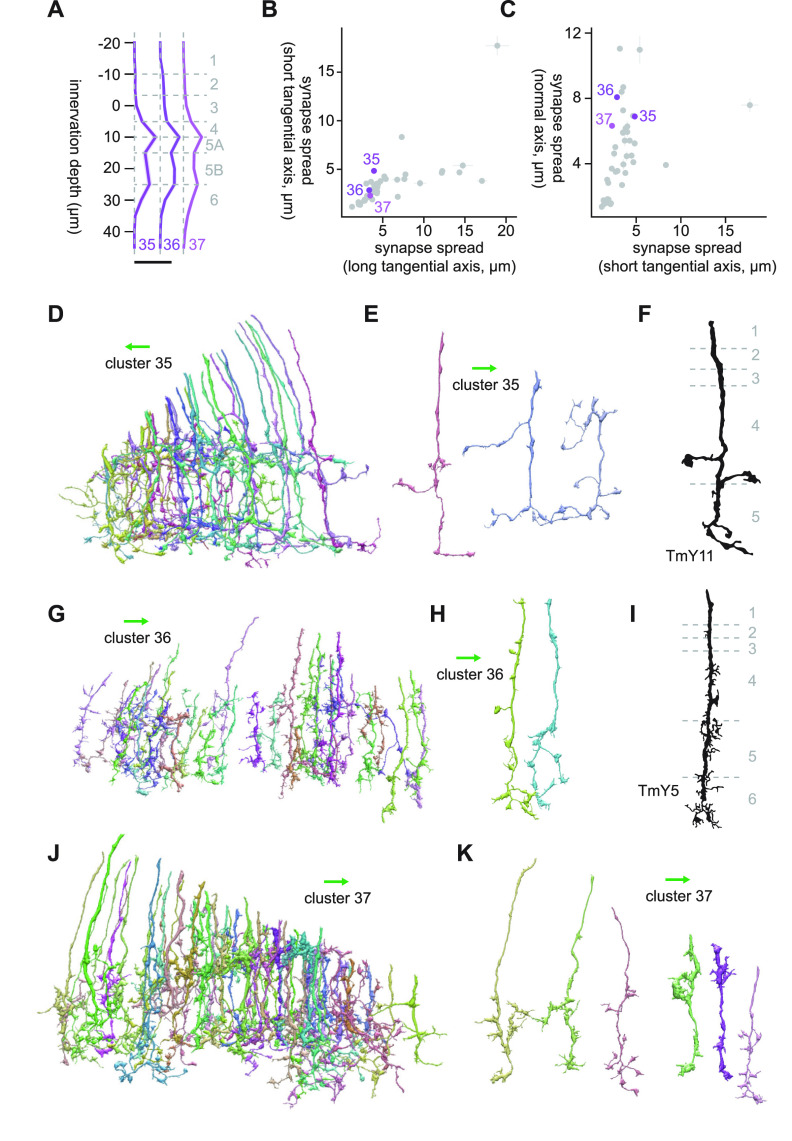
Morphology of clusters 35 through 37. ***A***, Mean innervation depth and (***B***, ***C***) synapse spread along the three axes of clusters 35 through 37. Error bars indicate SEM. ***A***, Vertical dotted line indicates zero synapses. The thick horizontal bar indicates 20 synapses. ***D***, A population of cluster 35 cells. ***E***, Representative examples of cluster 35 cells. ***F***, Morphology of a TmY11 axon terminal, from Fischbach and Dittrich (1989). ***G***, A population of cluster 36 cells. ***H***, Representative examples of cluster 36 cells. ***I***, Morphology of a TmY5 axon terminal, from Fischbach and Dittrich (1989). ***J***, A population of cluster 37 cells. ***K***, Examples cluster 37 cells. The green arrows indicate the anterior direction.

Clusters 35 through 37 were another set of vertically diffuse clusters (Fig. 18*A*), which on average appeared to be bistratified in Lo4/5A and Lo5B/6, but the pattern was less obvious compared with other bistratified clusters. The major postsynaptic targets of cluster 35 were Li16 (7.0 synapses/cell), Li12 (6.1 synapses/cell), LC15 (2.8 synapses/cell), LC10 (1.3 synapses/cell), and Li19 (1.1 synapses/cell). The dominant cell type of cluster 35 had branches sticking out of the main neurite perpendicularly, which aligned with the short tangential axis (Fig. 18*D*,*E*). This morphology strongly resembled that of TmY11 (Fischbach and Dittrich, 1989; Fig. 18*F*). The major postsynaptic targets of cluster 36 were LC20 (6.3 synapses/cell), Li12 (2.3 synapses/cell), LC22 (1.2 synapses/cell), LC10 (1.2 synapses/cell), and Li19 (1.0 synapses/cell). The dominant cell type of this cluster appeared more vertically diffuse than cluster 35 (Fig. 18*G*,*H*), with many short protrusions along its main neurite ranging from L4 to L6. The best morphologic match we could find for this cluster was TmY5 (Fischbach and Dittrich, 1989; Fig. 18*I*). Cluster 37 was another large, heterogeneous cluster consisting of a variety of vertically diffuse neurons resembling cells from clusters 32 through 36 (Fig. 18*J*,*K*). Similar to cluster 25, cluster 37 contained many fragments around the dent in the sample. The postsynaptic cell types with more than a single synapse per cell were LC10 (1.8 synapses/cell) and LC20 (1.4 synapses/cell).

#### Clusters 38 through 40

**Figure 19.**
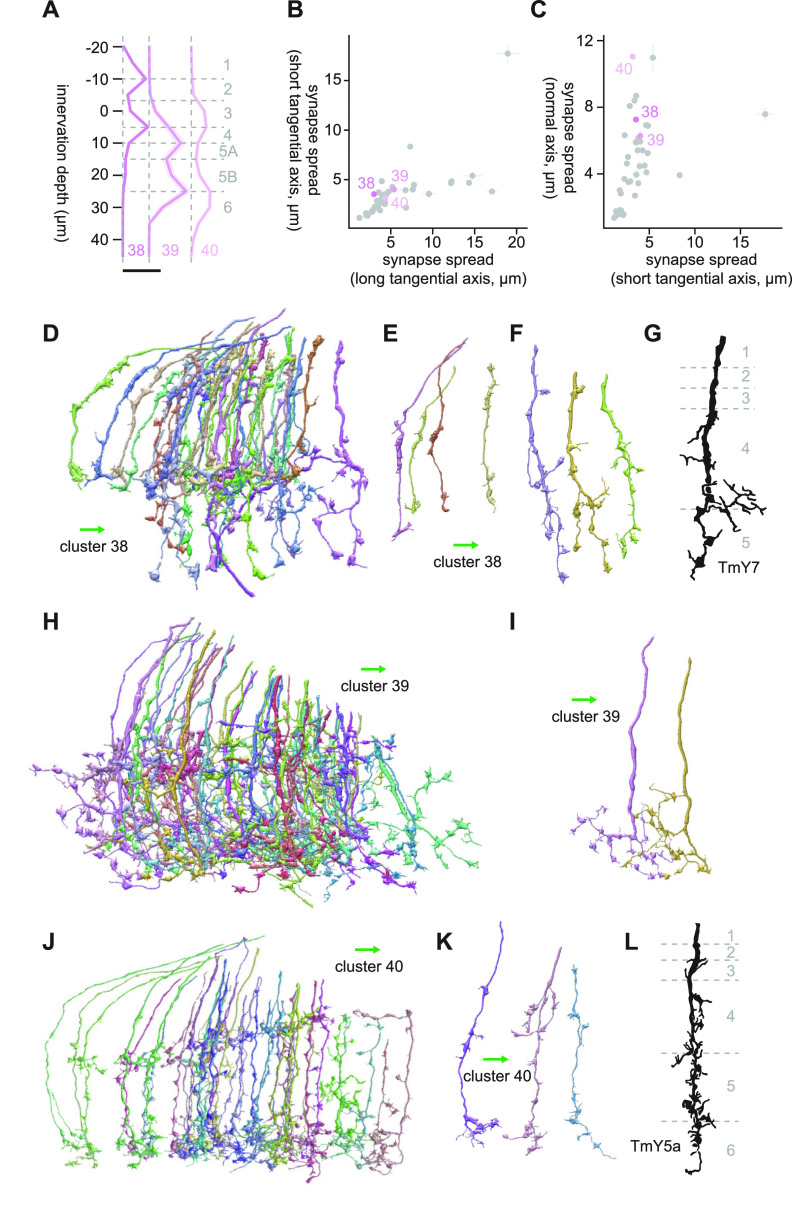
Morphology of clusters 38 through 40. ***A***, Mean innervation depth and (***B***, ***C***) synapse spread along the three axes of clusters 38 through 40. Error bars indicate SEM. ***A***, Vertical dotted line indicates zero synapses. The thick horizontal bar indicates 20 synapses. ***D***, A population of cluster 38 cells. ***E***, ***F***, Representative examples of two cell types included in cluster 38. Cells in ***E*** resembled cluster 6. ***G***, Morphology of a TmY7 axon terminal, from Fischbach and Dittrich (1989). ***H***, The entire population of cluster 39 cells. ***I***, Representative examples of cluster 39 cells. ***J***, A population of cluster 40 cells. ***K***, Representative examples of cluster 40 cells. ***L***, Morphology of a TmY5a axon terminal, from Fischbach and Dittrich (1989). The green arrows indicate the anterior direction.

Clusters 38 through 40 were vertically diffuse clusters with shallower layer innervations (Fig. 19*A*). Cluster 38 mainly innervated Lo1/2 and Lo3/4 (Fig. 19*A*). Their major postsynaptic targets were LC4 (4.8 synapses/cell), Li17 (3.9 synapses/cell), mALC1 (3.4 synapses/cell), LPLC1 (1.9 synapses/cell), and LC12 (1.6 synapses/cell). Morphologically, cluster 38 appeared to contain at least two dominant cell types, which is likely why this cluster showed up as two distant clusters on the UMAP embedding (Fig. 3*E*). The first cell type was a short, vertical cell with Y-shaped ending at L4 and swelling in a shallower layer (Fig. 19*D*,*E*). This morphology, as well as its connectivity, made it resemble cells in cluster 6, which we guessed to be either Tm4 or TmY2 (Fig. 7*G–I*). The continuity of clusters 6 and 38 can be also seen from the UMAP embedding (Fig. 3*E*). The other cell type in cluster 38 reached a deeper layer, likely Lo5A (Fig. 7*F*), and had a knobby appearance with more branching than the first cell type. The best morphologic match we could find for this cell type was TmY7 (Fischbach and Dittrich, 1989; Fig. 19*G*).

Cluster 39 was a small cluster of homogenous cells innervating only the ventral rim of the lobula (Fig. 19*H*). These cells had branching terminals with knobby endings (Fig. 19*I*). The major postsynaptic targets of this cluster were Li14 (13.1 synapses/cell), LC10 (7.3 synapses/cell), PS179 (3.9 synapses/cell), LPLC2 (3.3 synapses/cell), and LC14 (2.7 synapses/cell). Extensive connectivity to Li14 as well as PS179 made this cluster unique. It is unclear whether this cluster constitutes a retinotopically specialized cell type by itself. Alternatively, this could be a subset of a cell type whose other cells are lost in large clusters like cluster 37, clustered separately because of errors in innervation depth estimation caused by high curvature of the lobula layers at the rim.

Cluster 40 was the most vertically diffuse cluster with the synapse spread of 11.0 μm along the normal axis (Fig. 19*C*). This cluster was a homogeneous cluster of bistratified cells in Lo3/4 and Lo6, with minor protrusions in the layers in between (Fig. 19*A*,*J*,*K*). The major postsynaptic targets of this cluster were LC10 (4.3 synapses/cell), Li11 (3.1 synapses/cell), LPLC2 (2.2 synapses/cell), LT51 (2.2 synapses/cell), and LC4 (1.8 synapses/cell). The morphology of these cells resembled that of TmY5a (Fischbach and Dittrich, 1989; Davis et al., 2020; Fig. 19*K*,*L*), for example, although we are not particularly confident about this identification.

## Discussion

The lobula is one of the deepest neuropils in the fly visual system and is thought to detect behaviorally relevant visual features and send outputs to the central brain (Otsuna and Ito, 2006; Mu et al., 2012; Wu et al., 2016). While a number of cell types providing inputs to and sending outputs from the lobula have been characterized, connectivity among them, critical information to understanding the computations taking place in the lobula, have been largely missing. In the present study, we attempted to fill this gap by performing connectivity-based and morphology-based clustering on fragmented lobula axon terminals in the hemibrain dataset (Scheffer et al., 2020). Among the 40 clusters, we were able to find some morphologically matching cell types for 24 clusters, with varying degrees of confidence (Table 1). The cell types we identified with most confidence were as following: T3 (clusters 1 through 4), T2 (cluster 5), TmY29 and/or Tm28 (cluster 10), Tm20 (cluster 17), Tm9 (cluster 30), and TmY11 (cluster 35). These were mostly cell types that had distinctive terminal morphology in the lobula (Fischbach and Dittrich, 1989). The other identifications, Tm4 and/or TmY2 (clusters 6 and 38), Tm19 (clusters 12, 13), Tm11 (cluster 16), TmY10 (cluster 19), Li1 (cluster 20), Tm8 (cluster 21), T2a and/or Tm21 (cluster 23), Tm5Y (clusters 26 and 32), Tm5Y (cluster 32), Tm5 (clusters 33 and 34), TmY5 (cluster 36), TmY5a (cluster 40), were more speculative.

The current dataset lacks the information required to definitively identify cell types: the innervation patterns in the medulla and lobula plate. Future connectomic reconstructions encompassing the entire optic lobe will be necessary to unambiguously identify the putative cell types identified here. This can be in principle done by tracing in the FAFB EM volume (Zheng et al., 2018) on the FlyWire website (Dorkenwald et al., 2021). An alternative approach is to use molecular tools to map connectivity among genetically defined cell types, such as trans-synaptic GFP reconstitution (GRASP; Feinberg et al., 2008; Shearin et al., 2018), trans-Tango (Talay et al., 2017), and optogenetics (Simpson and Looger, 2018). This approach requires generating or identifying selective transgenic drivers to target new Tm and TmY neurons, and will be required to define functional connectivity, rather than anatomic connectivity. Neuroanatomical tools such as color depth maximum intensity projection mask search (Otsuna et al., 2018; Clements et al., 2020b) as well as marker gene identification with single cell RNA sequencing approaches (Özel et al., 2021) will be useful to this end. We found clusters consisting of apparently homogeneous cells whose morphology did not match any previously reported Tm or TmY cells (Fischbach and Dittrich, 1989; clusters 8, 9, 18, 22, 27–29, 39), likely representing new cell types. Given the existence of these novel neuron types as well as some mixture clusters, the 40 clusters we chose is likely smaller than the true number of Tm and TmY types.

While most of the putative cell types identified here have never been studied physiologically, there are some exceptions. For example, Tm4 (clusters 6/38) and Tm9 (cluster 30) are OFF cells presynaptic to T5 (Shinomiya et al., 2014; Serbe et al., 2016). Tm5 (clusters 33/34) and Tm20 (cluster 17) are spectrally sensitive cells downstream of the inner photoreceptors (Gao et al., 2008; Melnattur et al., 2014; Lin et al., 2016). T2 (cluster 5) and T3 (clusters 1–4) are known LC/LPLC inputs with selectivity for small moving objects (Keleş et al., 2020; Tanaka and Clark, 2020, 2021). Interestingly, the spectrally sensitive Tm neurons and object selective neurons appeared to converge on several LC neurons. For example, both LC11 and LC15 received sizable inputs from putative T3 and Tm5, suggesting that these cell types may prefer objects of specific colors. For lobula input neurons without known physiology, their connectivity to previously characterized LC/LPLCs can hint at their function. For example, cluster 32 neurons (putative Tm5Y) provide sizable inputs to LC6, 9, 13 and LPLC1, 2. LC6 (Wu et al., 2016; Morimoto et al., 2020), LPLC1 (Tanaka and Clark, 2021), and LPLC2 (Klapoetke et al., 2017; Ache et al., 2019) are sensitive to looming stimuli, suggesting that Tm5Y might play an important role in loom detection. Another example is cluster 10 neurons (TmY9/Tm28), which selectively connect to LC15. LC15 responds preferentially to moving, narrow vertical bars (Städele et al., 2020). The highly anisotropic dendrites of TmY9/Tm28 oriented along the dorsoventral axis (Fischbach and Dittrich, 1989) could be well suited to pool excitation along the vertical visual axis to detect long bars. Overall, the analysis presented here advances our knowledge of lobula connectivity and generates testable hypotheses for how lobula visual projection neurons become selective for specific visual features.

## Synthesis

Reviewing Editor: David Schoppik, New York University - Langone Medical Center Decisions are customarily a result of the Reviewing Editor and the peer reviewers coming together and discussing their recommendations until a consensus is reached. When revisions are invited, a fact-based synthesis statement explaining their decision and outlining what is needed to prepare a revision will be listed below. The following reviewer(s) agreed to reveal their identity: NONE.

The lobula of the Drosophila optic lobe is poised to be a key processing step in extracting behaviorally relevant features from an animal’s environment. However, our current understanding of its connectivity is rather limited, representing a missed opportunity to understand how feature extracting circuits are assembled and how their connectivity contributes to feature computations. Here, the authors develop a clustering-based analysis to identify cell types and map connectivity within the lobula, circumventing a limitation of the Drosophila hemibrain dataset that does not capture the full morphology of lobula neurons. The work in this paper is valuable in that it provides a method for relatively rapid cell type classification that can be used by other researchers dealing with large EM datasets. The findings of the paper also provide initial connectivity maps for the lobula that can be further evaluated with functional experiments.

Major suggestions

1. The Results text section of the paper is very limited. The information contained in the appendix (“detailed characterization of the 40 clusters”) is extremely helpful. Please consider moving this text into the Results -- 2/2 fly neuroscientists asked would prefer a proper “reference” paper rather than having this useful information buried in an Appendix.

2. Figure 1 - please display the relative size of LT1, the neuron that is being used to create a coordinate system for all of the lobula neurons in this study, as compared to individual lobula neurons (ex. Tm3)?

3. Similarly, the PCs for LT1 are actually based on the synapse locations across all three LT1 - is there a more accurate way to represent this in Figure 1B?

4. Line 94 in materials and methods - what was the reason for using the 50-500 synapse criteria?

5. Line 159 results - “The patterns of innervation captured by the two methodologies agreed well” - if the layers can be well identified across the two analysis methods, can this instead be quantified and compared?

6. Figure 1A designates layers 1,2,3,4, 5A, 5B and 6, but in figure 1B the layers are not labeled and the dotted lines are missing a layer from 1A.

Minor suggestions

1. Throughout the text, “lobula” instead of “the lobula” is used. Please use “the” to make it easier to read the text. Ex, lines 5-7 of the abstract, line 53, etc.

2. Additionally, the line 5-7 sentence is awkward, see suggestions: “However, connectomic analyses of (the) lobula, a putative (brain region for) object-like feature detection, remains underdeveloped, largely because of incomplete data on the inputs to the brain region.”

3. Line 61 “putative (input) cell types"

4. In the methods, the cluster number was set to 40 (line 138). As the EM datasets have revealed cell types not described in Fischbach and Dittrich (1989), this is likely an underrepresentation of the number of cell types in the lobula - this limitation should be discussed.

## Author Response

Dear reviewers,

We appreciate your helpful suggestions to improve the manuscript.

We have edited our manuscript to address your concerns. The point-by-point replies to the concerns raised in the reviews can be found below.

We hope these revisions have made the revised manuscript suitable for publication.

Sincerely,

Damon Clark

-

Synthesis of Reviews:

Significance Statement Comments for Author (Required):

Your en dash should be an em dash

We replaced the en dash with em dash.

Comments on the Visual Abstract for Author (Required):

The work is too pretty not to have a visual abstract -- please consider making one.

We created a visual abstract for the paper.

Synthesis Statement for Author (Required):

The lobula of the Drosophila optic lobe is poised to be a key processing step in extracting behaviorally relevant features from an animal’s environment. However, our current understanding of its connectivity is rather limited, representing a missed opportunity to understand how feature extracting circuits are assembled and how their connectivity contributes to feature computations. Here, the authors develop a clustering-based analysis to identify cell types and map connectivity within the lobula, circumventing a limitation of the Drosophila hemibrain dataset that does not capture the full morphology of lobula neurons. The work in this paper is valuable in that it provides a method for relatively rapid cell type classification that can be used by other researchers dealing with large EM datasets. The findings of the paper also provide initial connectivity maps for the lobula that can be further evaluated with functional experiments.

Major suggestions

1. The Results text section of the paper is very limited. The information contained in the appendix (“detailed characterization of the 40 clusters”) is extremely helpful. Please consider moving this text into the Results -- 2/2 fly neuroscientists asked would prefer a proper “reference” paper rather than having this useful information buried in an Appendix.

Thank you for this comment. We have moved the text and figures that were previously included in the Appendix back into the Results section.

2. Figure 1 - please display the relative size of LT1, the neuron that is being used to create a coordinate system for all of the lobula neurons in this study, as compared to individual lobula neurons (ex. Tm3)?

Thank you for the helpful suggestion. In the updated Figure 1, we included the morphology of reconstructed LT1 neurons, alongside with some “neurons of interest” from the cluster 1 (putative T3s) to give a better sense of scale. We also added a visualization of the parabolic fit to the LT1 synapses (Fig. 1E).

3. Similarly, the PCs for LT1 are actually based on the synapse locations across all three LT1 - is there a more accurate way to represent this in Figure 1B?

We updated the corresponding panels of Fig. 1B to show multiple LT1 neurons in different colors. In doing so, we realized there are actually four, rather than three, instances of LT1 neurons in the hemibrain. We updated this in the Methods section.

4. Line 94 in materials and methods - what was the reason for using the 50-500 synapse criteria?

For the lower bound, fragments less than 50 synaptic sites (which are numerous) were difficult to reliably classify due to the small amount of connectivity data available. The upper bound of 500 synapses is close to the size of relatively small amacrine-like cells in the lobula (e.g., Li13) or the smallest LCs (e. g., LC12). We expected our neurons of interest (mainly Tm and TmY) to be smaller than them. We have added this reasoning to the updated Methods section.

Also, in revising the manuscript, we came to realize that we were counting the total number of synaptic sites rather than synapses for deciding which neurons to include in our analysis (presynaptic sites typically have multiple synapses). We corrected this point in the updated Methods section. This change does not affect our interpretation of data.

5. Line 159 results - “The patterns of innervation captured by the two methodologies agreed well” - if the layers can be well identified across the two analysis methods, can this instead be quantified and compared?

Thank you for the helpful suggestion. The approximate layer boundaries we drew in Fig. 2B are based on its (qualitative) comparison to the figures in Wu et al. 2016, and we do not have a solid independent basis to classify hemibrain synapses into layers right now. The data from Wu et al. 2016 is also rather qualitative, making the suggested quantitative comparison difficult. We highlight the approximate nature of the layer boundaries in the updated figure caption.

6. Figure 1A designates layers 1,2,3,4, 5A, 5B and 6, but in figure 1B the layers are not labeled and the dotted lines are missing a layer from 1A.

We fixed these errors.

Minor suggestions

1. Throughout the text, “lobula” instead of “the lobula” is used. Please use “the” to make it easier to read the text. Ex, lines 5-7 of the abstract, line 53, etc.

Thank you for the suggestion. In the updated manuscript we refer it as “the lobula” consistently.

2. Additionally, the line 5-7 sentence is awkward, see suggestions: “However, connectomic analyses of (the) lobula, a putative (brain region for) object-like feature detection, remains underdeveloped, largely because of incomplete data on the inputs to the brain region.”

We paraphrased it to “However, connectomic analyses of the lobula, a neuropil putatively dedicated to detecting object-like features,...”. Thank you for the suggestion.

3. Line 61 “putative (input) cell types"

The typographical error is fixed.

4. In the methods, the cluster number was set to 40 (line 138). As the EM datasets have revealed cell types not described in Fischbach and Dittrich (1989), this is likely an underrepresentation of the number of cell types in the lobula - this limitation should be discussed.

We agree with the reviewer that the number 40 is likely smaller than the true number of Tm and TmY cell types. In the updated manuscript, we acknowledge this limitation in the Discussions (line 671).
